# Indices for dynamic evaluation of indoor humidity and thermal environment

**DOI:** 10.1038/s44172-023-00109-9

**Published:** 2023-08-19

**Authors:** Jiale Hu, Yingying Wang, Dengjia Wang, Hu Du, Jianhua Fan, Yanfeng Liu, Xin Sun

**Affiliations:** 1 School of Building Services Science and Engineering, Xi’an University of Architecture and Technology, Xi’an, China; 2 State Key Laboratory of Green Building, Xi’an University of Architecture and Technology, Xi’an, China; 3 School of Civil Engineering and Built Environment, Liverpool John Moores University, Liverpool, L3 3AF UK; 4 Department of Civil Engineering, Technical University of Denmark, Brovej 118Kgs., Lyngby, DK2800 Denmark

**Keywords:** Civil engineering, Environmental impact

## Abstract

Moisture sources release wet-components into indoor air, affecting the occupants’ health, air conditioning energy consumption, and building service-life. Wet-component evaporation and diffusion are dynamic processes, and yet existing indices are limited in their ability to accurately describe moisture sources dynamically influencing indoor air. Here we propose two indices CRI^t^_(H),_ an index of the rate of humidity contribution change, and CRI^t^_(c)_ as the rate of indoor climate contribution change. Taking a humidifier as the source, we use our indices to compare by experiment the impact of source parameters on a variety of ambient conditions over space and time. Our approach accurately reflects how the moisture source affect humidity and temperature, with identification of specific stages of dynamic influence. This study will be beneficial for the establishment of transient indoor environmental models, regulation of air-conditioning systems, and sustainable control of the indoor environment.

## Abstract (web summary)

Existing indices available to quantify the influence of moisture sources on air humidity and temperature are limited in their ability to accurately describe dynamic phenomena. Jiale Hu and colleagues propose two dynamic indices and use them to experimentally evaluate the dynamic indoor environment change performance of a humidifier.

## Introduction

**Fig. 1:**
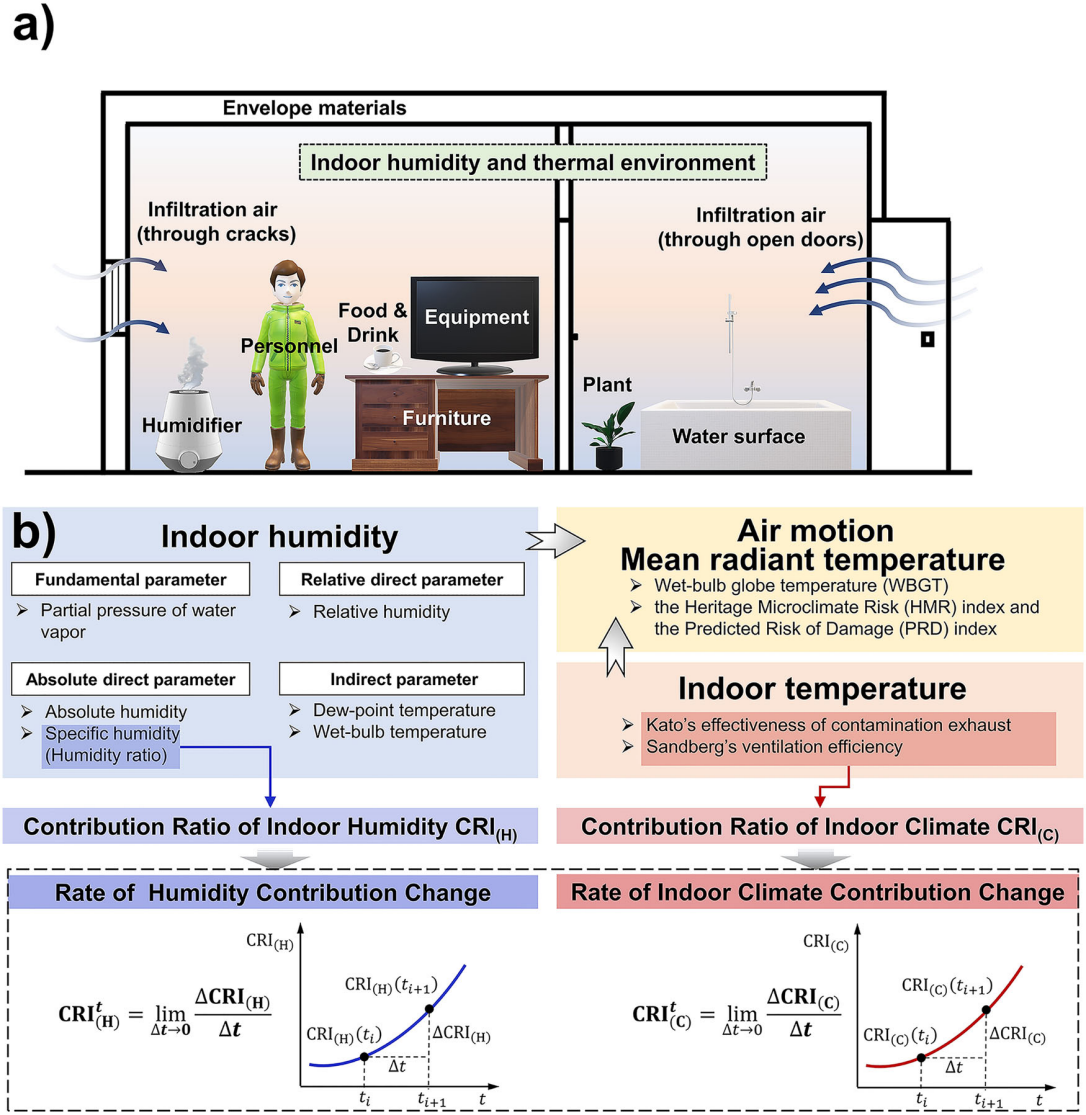
Concept illustration. **a** Indoor moisture sources. **b** Indices for indoor humidity and thermal environment evaluation.

There exist kinds of indoor moisture sources (Fig. 1a), such as personnel^1^, equipment^2^, envelope materials, and plants, which absorb wet-component (water droplets and water vapor) from or release it to the air at different rates, thus changing the indoor environment. The absorption or release intensity and the gas-liquid ratio of wet-component are considerably different among sources. For example, the moisture release rate of the human is 30 ~ 300 g h^−1^ under different exercise intensities, while that of plants^3^ is only 0.84 ~ 20.00 g h^−1^. In addition, larger plants produce more water vapor^4^. When a source continuously releases large amounts of wet-components, humidity increases rapidly, which can produce dew and mold on the walls^5,6^, and cause respiratory discomfort and allergies^7–9^. When the humidity is overly low (≤30%) due to the sources absorbing wet-components, dryness will not only affect the thermal comfort of occupants^10^ but also cause respiratory pain^11,12^, eye itching^13–15^ and static electricity. Moreover, overly high and low air humidity may favor the transmission and survival of some viruses^16–19^. Accordingly, reasonable indicators, which can accurately depict the influence of moisture sources on the indoor environment, will be beneficial for regulating air-conditioning with lower energy consumption and providing a satisfying environment for personnel.

The existing indexes for quantifying the influence of moisture sources on air humidity (Fig. 1b) can be divided into fundamental parameters (partial pressure of water vapor), absolute direct parameters (absolute humidity, specific humidity (humidity ratio)), relative direct parameters (relative humidity (RH)), and indirect parameters (dew-point temperature, wet-bulb temperature)^20^. To describe the effect of different objects on the humidity environment, scholars have modified these indexes to make them suitable for corresponding problems. Yanagi et al.^21^ investigated the impact of humidity on microbial contamination by employing the cumulative ratio of the average RH. In the indoor wall condensation model established by Ma et al.^22^, transient accessibility indices based on humidity ratio are used to predict the transient distribution of wet-components. Teodosiu^23^ simulated the thermo-solution-convection and condensation of humid-air on the surface, in which water-vapor mass fraction was used. Lucero-Gómez et al.^24^ evaluated the humidity environment with high control requirements by calculating the climatic excursions of RH fluctuation, and put forward efficient maintenance measures for air-conditioning. In addition, researchers also proposed comprehensive indices to assess the indoor environment by adding other parameters, such as air temperature, air motion and mean radiant temperature. Gao et al.^25^ established a simplified indoor wet-bulb globe temperature (WBGT) formula and analyzed its relationship with RH, which can be used to evaluate the indoor environment of naturally ventilated buildings. Bonora et al.^26^ established two indoor microclimate indicators based on air temperature and RH: the Heritage Microclimate Risk (HMR) index and the Predicted Risk of Damage (PRD) index, to determine the risk level of the indoor environment for cultural heritage.

However, the air temperature and specific humidity are objective parameters. To evaluate the impact of indoor real or virtual heat sources on temperature distribution, Kato et al.^27^ proposed the Contribution Ratio of Indoor Climate (${{{{{{\rm{CRI}}}}}}}_{\left({{{{{\rm{C}}}}}}\right)}$) based on Kato’s effectiveness of contamination exhaust^28^ and Sandberg’s ventilation efficiency^29^. This indicator can quantify the diffusion process of indoor heat sources and their effect on the air temperature. To thoroughly analyze the impact mechanism of indoor moisture sources on the humidity environment, Huang et al.^30^, referring to the establishment of ${{{{{{\rm{CRI}}}}}}}_{\left({{{{{\rm{C}}}}}}\right)}$, proposed Contribution Ratio of Indoor Humidity ${{{{{{\rm{CRI}}}}}}}_{\left({{{{{\rm{H}}}}}}\right)}$ based on specific humidity. Due to the effectiveness of ${{{{{{\rm{CRI}}}}}}}_{\left({{{{{\rm{H}}}}}}\right)}$ in detailed humidity environment design^29^, Huang et al. used it to simulate the humidity field and combined it with the genetic algorithm to establish an efficient optimization design system^31^. In addition, Zhu et al.^32^ realized the rapid prediction of indoor humidity by combining ${{{{{{\rm{CRI}}}}}}}_{\left({{{{{\rm{H}}}}}}\right)}$ with a low-dimensional linear humidity model and optimized the balance between the personal perception of humidity and air-conditioning humidity loads. Nevertheless, ${{{{{{\rm{CRI}}}}}}}_{\left({{{{{\rm{C}}}}}}\right)}$ and ${{{{{{\rm{CRI}}}}}}}_{\left({{{{{\rm{H}}}}}}\right)}$, the spatial description indexes of the sources affecting the indoor environment at a certain moment, have limitations in reflecting the dynamic characteristics of transient evaporation and diffusion of wet-components produced by the source influencing the indoor temperature and humidity.

Here, we propose a index: the Rate of Humidity Contribution Change (${{{{{{\rm{CRI}}}}}}}_{({{{{{\rm{H}}}}}})}^{{{{{{\rm{t}}}}}}}$) through transient treatment of ${{{{{{\rm{CRI}}}}}}}_{\left({{{{{\rm{H}}}}}}\right)}$, which represents the variation of ${{{{{{\rm{CRI}}}}}}}_{\left({{{{{\rm{H}}}}}}\right)}$ at a space point per unit time. Considering the evaporation and diffusion of the wet-component also lead to its heat exchange with indoor air, then affecting the air temperature field, ${{{{{{\rm{CRI}}}}}}}_{\left({{{{{\rm{C}}}}}}\right)}$ is also transiently processed into the Rate of Indoor Climate Contribution Change (${{{{{{\rm{CRI}}}}}}}_{({{{{{\rm{C}}}}}})}^{{{{{{\rm{t}}}}}}}$)^33^. Subsequently, the feasibility of ${{{{{{\rm{CRI}}}}}}}_{({{{{{\rm{H}}}}}})}^{{{{{{\rm{t}}}}}}}$ and ${{{{{{\rm{CRI}}}}}}}_{({{{{{\rm{C}}}}}})}^{{{{{{\rm{t}}}}}}}$ in describing the source influencing indoor air dynamically is verified by analyzing the variation of two indexes, which were computed from the indoor environmental parameters influenced by an ultrasonic humidifier under various source parameters and environmental conditions. The results show that the variation of air humidity under the influence of a moisture source can be divided into an increase stage and a stable stage, while that of air temperature under the influence of it as a heat source can be divided into a decrease stage and an increase stage. Based on the variation of ${{{{{{\rm{CRI}}}}}}}_{({{{{{\rm{H}}}}}})}^{{{{{{\rm{t}}}}}}}$ and ${{{{{{\rm{CRI}}}}}}}_{({{{{{\rm{C}}}}}})}^{{{{{{\rm{t}}}}}}}$, we found that the dynamical influence of the source on the indoor environment is different for various source parameters and environmental conditions.

## Results and Discussion

### ${{{{{{\bf{CRI}}}}}}}_{{{{{{\boldsymbol{(}}}}}}{{{{{\bf{H}}}}}}{{{{{\boldsymbol{)}}}}}}}^{{{{{{\bf{t}}}}}}}$, ${{{{{{\bf{CRI}}}}}}}_{\left({{{{{\bf{H}}}}}}\right)}$ and Air Humidity

**Fig. 2:**
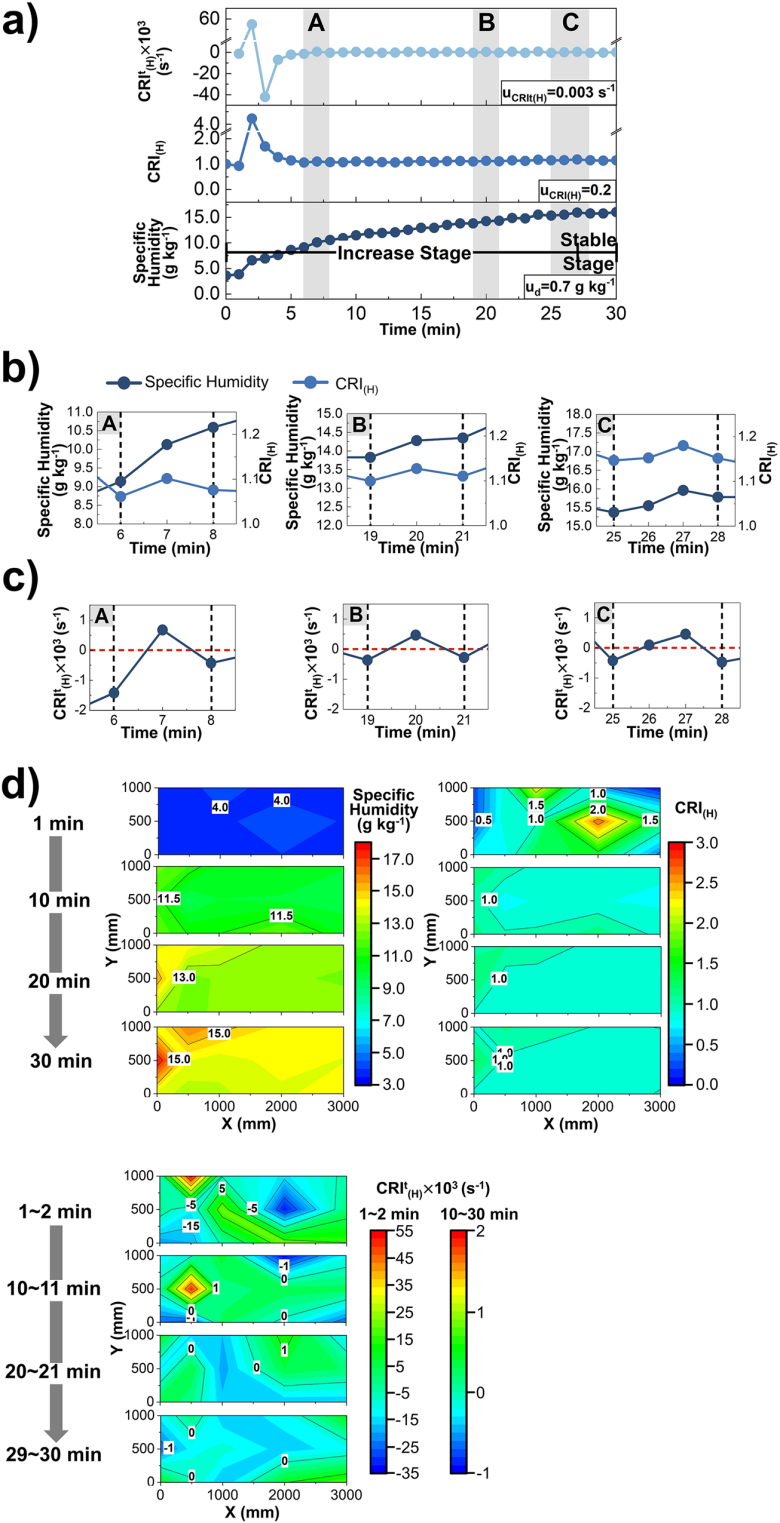
${{{{{{\rm{CRI}}}}}}}_{({{{{{\rm{H}}}}}})}^{{{{{{\rm{t}}}}}}}$, ${{{{{{\rm{CRI}}}}}}}_{\left({{{{{\rm{H}}}}}}\right)}$ and Air Humidity. **a** The variation of ${{{{{{\rm{CRI}}}}}}}_{({{{{{\rm{H}}}}}})}^{{{{{{\rm{t}}}}}}}$ (light blue), ${{{{{{\rm{CRI}}}}}}}_{\left({{{{{\rm{H}}}}}}\right)}$ (blue) and specific humidity (navy blue) at point (X,Y) = (500,1000) mm, whose uncertainty is u_CRIt(H)_, u_CRI(H)_ and u_d_ (*n* = 151 samples). **b** The variation of specific humidity (navy blue) and ${{{{{{\rm{CRI}}}}}}}_{\left({{{{{\rm{H}}}}}}\right)}$ (blue) during fluctuation cycle A(t = 6 ~ 8 min), B(t = 19 ~ 21 min), C(t = 25 ~ 28 min). **c** The variation of ${{{{{{\rm{CRI}}}}}}}_{({{{{{\rm{H}}}}}})}^{{{{{{\rm{t}}}}}}}$ (navy blue) during fluctuation cycle A(t = 6 ~ 8 min), B(t = 19 ~ 21 min), C(t = 25 ~ 28 min). **d** The spatial distribution of specific humidity and ${{{{{{\rm{CRI}}}}}}}_{\left({{{{{\rm{H}}}}}}\right)}$ at t = 1 min, 10 min, 20 min and 30 min, and ${{{{{{\rm{CRI}}}}}}}_{({{{{{\rm{H}}}}}})}^{{{{{{\rm{t}}}}}}}$ during t = 1 ~ 2 min, 10 ~ 11 min, 20 ~ 21 min, 29 ~ 30 min in the XOY plane (X = 0 ~ 3000 mm and Y = 0 ~ 1000 mm).

The variation of specific humidity, ${{{{{{\rm{CRI}}}}}}}_{\left({{{{{\rm{H}}}}}}\right)}$ and ${{{{{{\rm{CRI}}}}}}}_{({{{{{\rm{H}}}}}})}^{{{{{{\rm{t}}}}}}}$ at one point (X, Y) = (500 mm, 1000 mm) during the humidifier operation are analyzed in Fig. 2. And the uncertainty of each quantity is presented in the corresponding figure. When the moisture source conveys the wet-component to the air at a fixed intensity, the specific humidity shows an increasing trend, but its increase rate declines over time (Fig. 2a). The reason is that the moisture difference between the source and the indoor air decreases due to the continuous diffusion of moisture. When the humidity is close to the saturation value corresponding to ambient temperature, it begins to fluctuate regularly. Therefore, the process of source influencing air humidity at a point can be divided into an increase stage and a stable stage (IS_H_ and SS_H_). The ${{{{{{\rm{CRI}}}}}}}_{\left({{{{{\rm{H}}}}}}\right)}$ rapidly increases to 4.2 at t = 2 min, which results from the wet-component reaching this point earlier than other spatial locations. Subsequently, with the continuous diffusion of wet components during t = 2 ~ 5 min, the indoor humidity of other points begins to increase, and ${{{{{{\rm{CRI}}}}}}}_{\left({{{{{\rm{H}}}}}}\right)}$ at point (X, Y) = (500 mm, 1000 mm) decreases notably. After that, its ${{{{{{\rm{CRI}}}}}}}_{\left({{{{{\rm{H}}}}}}\right)}$ increases slightly in the range of 1.0 ~ 1.2 since this point is closer to the source outlet than other points. The increase of specific humidity of fluctuation period C is about 0.9 g kg^−1^ lower than that of period A, and the fluctuation range of period C becomes slightly narrower (Fig. 2b and c).

The ${{{{{{\rm{CRI}}}}}}}_{({{{{{\rm{H}}}}}})}^{{{{{{\rm{t}}}}}}}$ begins to fluctuate periodically based on 0 after peaking at t = 2 min and t = 3 min, and its fluctuation range varies with time. As shown in Fig. 2c, for the fluctuation periods A (t = 6 ~ 8 min), B (t = 19 ~ 21 min) and C (t = 25 ~ 28 min), ${{{{{{\rm{CRI}}}}}}}_{({{{{{\rm{H}}}}}})}^{{{{{{\rm{t}}}}}}}$ all first increases from negative value to positive and then decreases to negative value again during every period, but their fluctuation ranges are quite different from each other. A positive or negative ${{{{{{\rm{CRI}}}}}}}_{({{{{{\rm{H}}}}}})}^{{{{{{\rm{t}}}}}}}$ indicates an increase or decrease in ${{{{{{\rm{CRI}}}}}}}_{\left({{{{{\rm{H}}}}}}\right)}$, and its absolute value indicates the amount of change in ${{{{{{\rm{CRI}}}}}}}_{\left({{{{{\rm{H}}}}}}\right)}$ during the unit time. Moreover, the ${{{{{{\rm{CRI}}}}}}}_{({{{{{\rm{H}}}}}})}^{{{{{{\rm{t}}}}}}}$ variation curve can not only reflect the changes of ${{{{{{\rm{CRI}}}}}}}_{({{{{{\rm{H}}}}}})}^{{{{{{\rm{t}}}}}}}$ itself, but the area, enclosed by it and $t={t}_{i}$, $t={t}_{i+1}$, time-axis, reflects the total amount of ${{{{{{\rm{CRI}}}}}}}_{\left({{{{{\rm{H}}}}}}\right)}$ changes during the study time $t={t}_{i}$ ~ ${t}_{i+1}$. Therefore, ${{{{{{\rm{CRI}}}}}}}_{({{{{{\rm{H}}}}}})}^{{{{{{\rm{t}}}}}}}$ can be directly used to investigate the difference of dynamic characteristics among different periods of the moisture source influencing the indoor humidity distribution.

To compare the influence of source on indoor humidity environment at different locations, the spatial distribution of specific humidity, ${{{{{{\rm{CRI}}}}}}}_{\left({{{{{\rm{H}}}}}}\right)}$ and ${{{{{{\rm{CRI}}}}}}}_{({{{{{\rm{H}}}}}})}^{{{{{{\rm{t}}}}}}}$ in the XOY plane (X = 0 ~ 3000 mm and Y = 0 ~ 1000 mm) are analyzed in Fig. 2d. During the initial stage (from t = 1 min to t = 10 min), there is a large humidity difference between the moisture source and the indoor air. The specific humidity of all spatial points on the XOY plane increase by an average of 6.9 g kg^−1^, and the variation range of ${{{{{{\rm{CRI}}}}}}}_{\left({{{{{\rm{H}}}}}}\right)}$ shrinks from 0.1 ~ 2.6 to 0.8 ~ 1.2, whose spatial distribution all become relatively uniform at t = 10 min. The spatial distribution of ${{{{{{\rm{CRI}}}}}}}_{({{{{{\rm{H}}}}}})}^{{{{{{\rm{t}}}}}}}$ is uneven, with the maximum value of 0.055 s^−1^ during t = 1 ~ 2 min and 0.002 s^−1^ during t = 10 ~ 11 min, which indicates the dynamic characteristics of indoor humidity variation is notable during this period. After the source is turned on for 20 min, the moisture difference between the source and the indoor air becomes small. The specific humidity begins to increase slowly or remains stable, and the ${{{{{{\rm{CRI}}}}}}}_{({{{{{\rm{H}}}}}})}^{{{{{{\rm{t}}}}}}}$ distribution tends to be more uniform. Noteworthy is that the specific humidity and ${{{{{{\rm{CRI}}}}}}}_{\left({{{{{\rm{H}}}}}}\right)}$ of spatial points located on the moisture flow trajectory are higher than other points due to they obtaining the wet-components more directly. At t = 30 min, higher specific humidity 17.7 g kg^−1^ and 16.0 g kg^−1^ appear at (X, Y) = (0 mm, 500 mm) and (500 mm, 1000 mm) respectively. The phenomenon at (X, Y) = (0 mm, 500 mm) is mainly due to the droplets with large particle size falling to the ground under the gravity, evaporating into water vapor and the vapor rising under the density difference. However, because of the interference of large velocity airflow at the source outlet ((X, Y) = (0 mm, 1000 mm)), the water vapor mainly gathers around the height of Y = 500 mm. The reason at (X, Y) = (500 mm, 1000 mm) may be that small and medium-sized droplets enter the chamber with an initial horizontal velocity and then evaporate and diffuse along the X-axis, while their velocity gradually decreases due to viscous forces. When the droplets reach X = 500 mm, they have a smaller horizontal velocity and a longer residence time, resulting in more water vapor generated by their evaporation. Subsequently, the dominant force in their motion process changes from inertial force to gravitational force, and most of the droplets fall in the form of a horizontal projectile motion.

Furthermore, since the ${{{{{{\rm{CRI}}}}}}}^{{{{{{\rm{t}}}}}}}$ is an instantaneous concept that is related to the variation in ${{{{{\rm{CRI}}}}}}$ and the time it takes to change, but not to the ${{{{{\rm{CRI}}}}}}$ value at one second, the ${{{{{\rm{CRI}}}}}}$ can be very small when the ${{{{{{\rm{CRI}}}}}}}^{{{{{{\rm{t}}}}}}}$ is large at the same point. In Fig. 2d, when the moisture flow produced by the source just entering the room, the ${{{{{{\rm{CRI}}}}}}}_{\left({{{{{\rm{H}}}}}}\right)}$ at (X, Y) = (500 mm, 1000 mm) is 0.9 at t = 1 min while ${{{{{{\rm{CRI}}}}}}}_{({{{{{\rm{H}}}}}})}^{{{{{{\rm{t}}}}}}}$ is as large as 0.055 s^−1^ during t = 1 ~ 2 min. On the other hand, the ${{{{{\rm{CRI}}}}}}$ can be large when ${{{{{{\rm{CRI}}}}}}}^{{{{{{\rm{t}}}}}}}$ is small, such as the ${{{{{{\rm{CRI}}}}}}}_{\left({{{{{\rm{H}}}}}}\right)}$ at t = 1 min and ${{{{{{\rm{CRI}}}}}}}_{({{{{{\rm{H}}}}}})}^{{{{{{\rm{t}}}}}}}$ during t = 1 ~ 2 min of (X, Y) = (2000mm, 500 mm).

### ${{{{{{\bf{CRI}}}}}}}_{{{{{{\boldsymbol{(}}}}}}{{{{{\bf{C}}}}}}{{{{{\boldsymbol{)}}}}}}}^{{{{{{\bf{t}}}}}}}$, ${{{{{{\bf{CRI}}}}}}}_{\left({{{{{\bf{C}}}}}}\right)}$ and Air Temperature

**Fig. 3:**
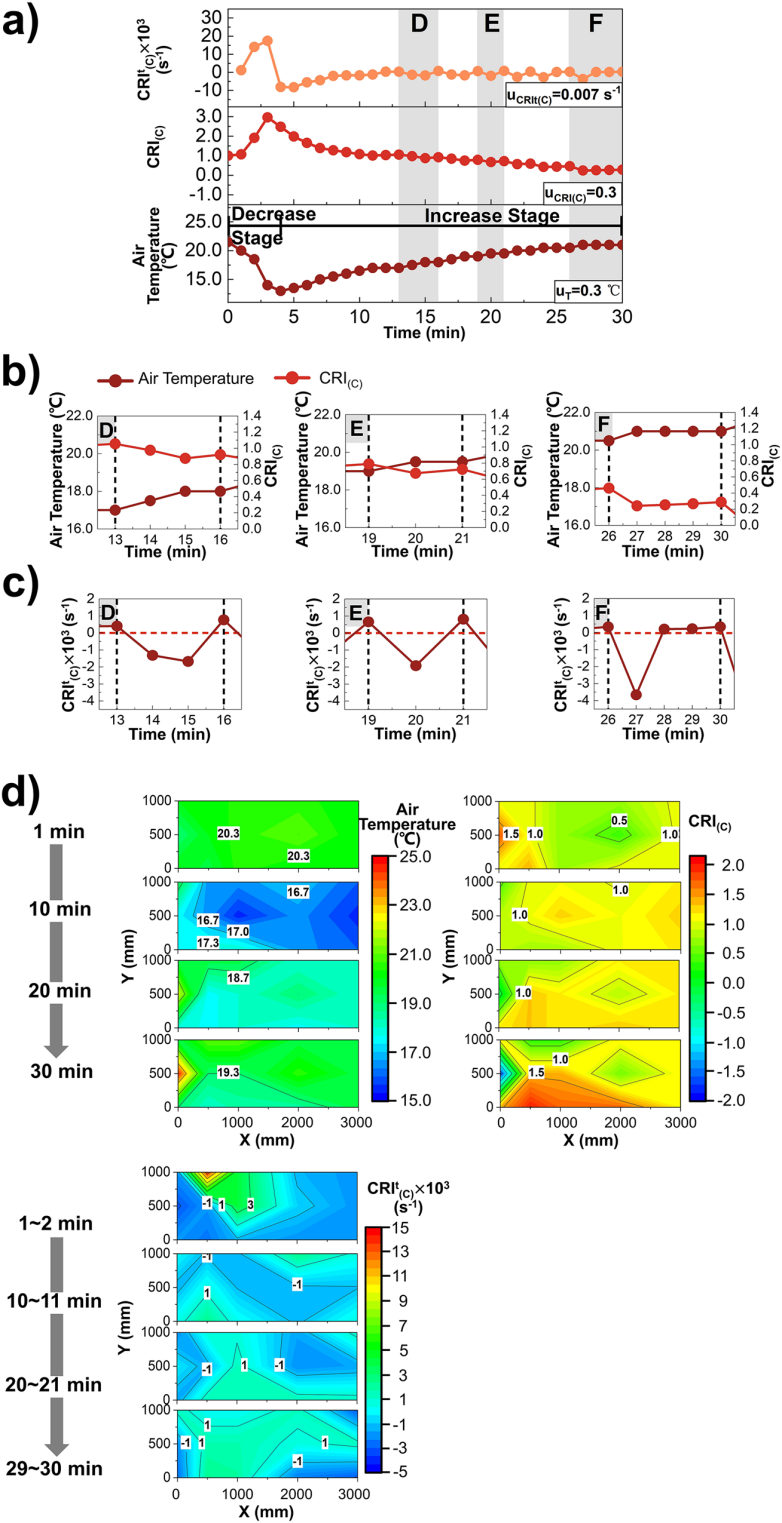
${{{{{{\rm{CRI}}}}}}}_{({{{{{\rm{C}}}}}})}^{{{{{{\rm{t}}}}}}}$, ${{{{{{\rm{CRI}}}}}}}_{\left({{{{{\rm{C}}}}}}\right)}$ and Air Temperature. **a** The variation of ${{{{{{\rm{CRI}}}}}}}_{({{{{{\rm{C}}}}}})}^{{{{{{\rm{t}}}}}}}$ (reddish orange), ${{{{{{\rm{CRI}}}}}}}_{\left({{{{{\rm{C}}}}}}\right)}$ (red) and air temperature (deep red) at point (X,Y) = (500,1000) mm, whose uncertainty is u_CRIt(C)_, u_CRI(C)_ and u_T_ (n = 151 samples). **b** The variation of air temperature (deep red) and ${{{{{{\rm{CRI}}}}}}}_{\left({{{{{\rm{C}}}}}}\right)}$ (red) during fluctuation cycle D(t = 13 ~ 16 min), E(t = 19 ~ 21 min), F(t = 26 ~ 30 min). **c** The variation of ${{{{{{\rm{CRI}}}}}}}_{({{{{{\rm{C}}}}}})}^{{{{{{\rm{t}}}}}}}$ (deep red) during fluctuation cycle D(t = 13 ~ 16 min), E(t = 19 ~ 21 min), F(t = 26 ~ 30 min). **d** The spatial distribution of air temperature and ${{{{{{\rm{CRI}}}}}}}_{\left({{{{{\rm{C}}}}}}\right)}$ at t = 1 min, 10 min, 20 min and 30 min, and ${{{{{{\rm{CRI}}}}}}}_{({{{{{\rm{C}}}}}})}^{{{{{{\rm{t}}}}}}}$ during t = 1 ~ 2 min, 10 ~ 11 min, 20 ~ 21 min and 29 ~ 30 min in the XOY plane (X = 0 ~ 3000 mm and Y = 0 ~ 1000 mm).

Due to the sensible and latent heat exchange between the moisture source and indoor air, the moisture source also affects air temperature while raising air humidity. The same point (X, Y) = (500 mm, 1000 mm) is chosen to analyze the variation process of air temperature, ${{{{{{\rm{CRI}}}}}}}_{({{{{{\rm{C}}}}}})}$ and ${{{{{{\rm{CRI}}}}}}}_{({{{{{\rm{C}}}}}})}^{{{{{{\rm{t}}}}}}}$ in Fig. 3.

Under this operating condition, the droplet temperature is larger than the air temperature, so the heat transfer process between the droplet and the air consists of the latent heat transfer of droplet evaporation, during which the droplet absorbs the heat of itself and the air, and the sensible heat transfer from the droplet to the air. When the source continuously feeds the wet components to the indoor air, the latent heat transfer for droplet evaporation is dominant at the initial stage due to the large humidity difference between the source and the air, leading to a rapid drop in the air temperature (Fig. 3a). As the air humidity increases, the mass transfer rate decreases and the evaporation process begin to slow down. When the latent heat exchange amount equals the sensible one, the air temperature reaches the lowest value of 13.0 °C. Subsequently, the sensible heat transfer from the moisture to the air becomes the dominant process, and the air temperature increases in a “ladder-like” pattern^34^. In this way, the dynamic influence process of the source on the air temperature can be divided into a decrease stage and an increase stage (DS_T_ and IS_T_). Since the air temperature is less than the initial value of 21.5 °C, the humidifier can be considered as a negative heat source for the indoor thermal environment. The significant change stage of ${{{{{{\rm{CRI}}}}}}}_{\left({{{{{\rm{C}}}}}}\right)}$ occurs during t = 0 ~ 7 min, which increases rapidly from 1.0 to 3.0 in the first 3 min and then decreases rapidly by 1.6 in the next 4 min. Subsequently, it gradually decreases and finally goes to 0.3 at t = 30 min.

${{{{{{\rm{CRI}}}}}}}_{({{{{{\rm{C}}}}}})}^{{{{{{\rm{t}}}}}}}$ first increases and then decreases during the DS_T_, followed by periodic fluctuations within IS_T_. As shown in Fig. 3b and Fig. 3c, during the fluctuation period D, the ${{{{{{\rm{CRI}}}}}}}_{({{{{{\rm{C}}}}}})}^{{{{{{\rm{t}}}}}}}$ at t = 14 min and 15 min is both negative and the absolute value of the latter one is larger, indicating that the ${{{{{{\rm{CRI}}}}}}}_{\left({{{{{\rm{C}}}}}}\right)}$ continues to decrease within t = 13 ~ 15 min and its decrease amount in unit time gradually increases. When t = 16 min, the ${{{{{{\rm{CRI}}}}}}}_{({{{{{\rm{C}}}}}})}^{{{{{{\rm{t}}}}}}}$ is a positive and small value, which suggests that ${{{{{{\rm{CRI}}}}}}}_{\left({{{{{\rm{C}}}}}}\right)}$ increases slightly during t = 15 ~ 16 min. The time proportion of ${{{{{{\rm{CRI}}}}}}}_{({{{{{\rm{C}}}}}})}^{{{{{{\rm{t}}}}}}}$ <0 in fluctuation periods E and F is smaller than that of period D, but the absolute value of ${{{{{{\rm{CRI}}}}}}}_{({{{{{\rm{C}}}}}})}^{{{{{{\rm{t}}}}}}}$ at t = 20 min and 27 min are higher than that at t = 15 min. The above phenomena show that during the IS_T_, the time proportion of decreasing ${{{{{{\rm{CRI}}}}}}}_{\left({{{{{\rm{C}}}}}}\right)}$ for the next fluctuation period will be smaller and the decrease amount of ${{{{{{\rm{CRI}}}}}}}_{\left({{{{{\rm{C}}}}}}\right)}$ per unit time will be larger than the current one. In addition, the time of ${{{{{{\rm{CRI}}}}}}}_{\left({{{{{\rm{C}}}}}}\right)}$ increasing will be longer and the increase amount of ${{{{{{\rm{CRI}}}}}}}_{\left({{{{{\rm{C}}}}}}\right)}$ at unit time will become smaller in the next fluctuation period.

Similar as above, a positive or negative ${{{{{{\rm{CRI}}}}}}}_{({{{{{\rm{C}}}}}})}^{{{{{{\rm{t}}}}}}}$ indicates an increase or decrease in ${{{{{{\rm{CRI}}}}}}}_{\left({{{{{\rm{C}}}}}}\right)}$, and its absolute value indicates the amount of change in ${{{{{{\rm{CRI}}}}}}}_{\left({{{{{\rm{C}}}}}}\right)}$ during unit time. Moreover, the ${{{{{{\rm{CRI}}}}}}}_{({{{{{\rm{C}}}}}})}^{{{{{{\rm{t}}}}}}}$ variation curve can not only reflect the changes of ${{{{{{\rm{CRI}}}}}}}_{({{{{{\rm{C}}}}}})}^{{{{{{\rm{t}}}}}}}$ itself, but the area, enclosed by it and $t={t}_{i}$, $t={t}_{i+1}$, time-axis, reflects the total amount of ${{{{{{\rm{CRI}}}}}}}_{\left({{{{{\rm{C}}}}}}\right)}$ changes during the study time $t={t}_{i}$ ~ ${t}_{i+1}$. Therefore, ${{{{{{\rm{CRI}}}}}}}_{({{{{{\rm{C}}}}}})}^{{{{{{\rm{t}}}}}}}$ can be directly used to investigate the difference of dynamic characteristics among different periods of the heat source influencing the air temperature distribution.

To compare the influence of source on indoor thermal environment at different locations, the spatial distribution of air temperature, ${{{{{{\rm{CRI}}}}}}}_{\left({{{{{\rm{C}}}}}}\right)}$ and ${{{{{{\rm{CRI}}}}}}}_{({{{{{\rm{C}}}}}})}^{{{{{{\rm{t}}}}}}}$ in the XOY plane (X = 0 ~ 3000 mm and Y = 0 ~ 1000 mm) are analyzed in Fig. 3d. When t = 1 min, the spatial distribution of air temperature and ${{{{{{\rm{CRI}}}}}}}_{\left({{{{{\rm{C}}}}}}\right)}$ is moderately uniform, while the maximum ${{{{{{\rm{CRI}}}}}}}_{({{{{{\rm{C}}}}}})}^{{{{{{\rm{t}}}}}}}$ is as high as 0.014 s^−1^ during t = 1 ~ 2 min at (X,Y) = (500 mm, 1000 mm). During the 10 min after the source is turned on, the droplet evaporation rate is large due to the large humidity difference between the source and the indoor air, and a large amount of heat is absorbed by the droplets from the indoor air simultaneously. The air temperature of all positions in XOY plane is reduced by 3.2 °C, and the spatial variation range of ${{{{{{\rm{CRI}}}}}}}_{\left({{{{{\rm{C}}}}}}\right)}$ narrows from 0.4 ~ 1.8 to 0.4 ~ 1.3. During t = 20 min~30 min, the points located on the moisture flow trajectory obtain the wet-components earlier, completing the DS_T_ and entering into the IS_T_ faster than other points, they have higher air temperature, lower ${{{{{{\rm{CRI}}}}}}}_{\left({{{{{\rm{C}}}}}}\right)}$.

### Comprehensive analysis of humidity and temperature

**Fig. 4:**
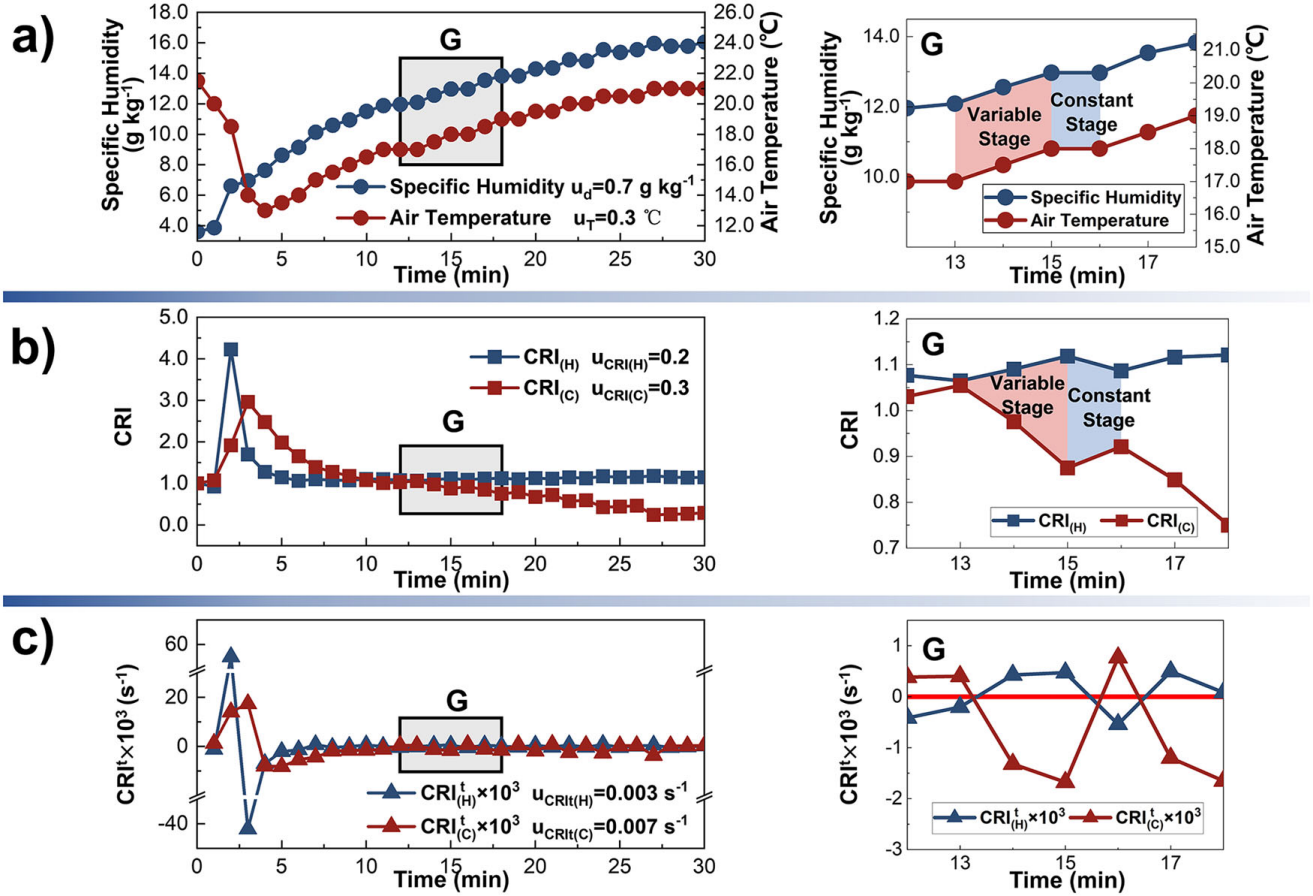
The relationship between the temperature and humidity. **a** Air specific humidity (navy blue curve with dots) and temperature (deep red with dots), whose uncertainty is u_d_ and u_T_. **b** Contribution Ratio of Indoor Humidity (${{{{{{\rm{CRI}}}}}}}_{\left({{{{{\rm{H}}}}}}\right)}$) (navy blue curve with squares) and Contribution Ratio of Indoor Climate (${{{{{{\rm{CRI}}}}}}}_{\left({{{{{\rm{C}}}}}}\right)}$) (deep red with squares), whose uncertainty is u_CRI(H)_ and u_CRI(C)_. **c** Rate of Humidity Contribution Change (${{{{{{\rm{CRI}}}}}}}_{({{{{{\rm{H}}}}}})}^{{{{{{\rm{t}}}}}}}$) (navy blue curve with triangles) and Rate of Indoor Climate Contribution Change (${{{{{{\rm{CRI}}}}}}}_{({{{{{\rm{C}}}}}})}^{{{{{{\rm{t}}}}}}}$) (deep red with triangles), whose uncertainty is u_CRIt(H)_ and u_CRIt(C)_.

The relationship between the air temperature and the humidity at (X, Y) = (500 mm, 1000 mm), affected by the source, is further shown in Fig. 4. In Fig. 4a, the air temperature decreases and then increases with time as the specific humidity increase continuously. When the humidity is just entering the IS_H_, the temperature decreases rapidly, indicating that the wet-component evaporation rate is much higher. About 8 min after the air temperature variation enters the IS_T_, the specific humidity starts to grow steadily. In the process of simultaneous increase of air temperature and humidity, the trends of the periodical variability of both and the duration of the variable stage and the constant stage in each cycle are essentially the same. The main reason is that when the wet component produced by the source reaches one position during the IS_H_ and IS_T_, it evaporates and then diffuses under the water vapor partial pressure difference, and concurrently the wet component transfers sensible heat to the air under temperature difference, which results in the simultaneous increase of air temperature and humidity. As the temperature and moisture differences decrease, the processes of heat exchange and water vapor diffusion nearly cease. As a result, the temperature and humidity remain constant. But when the new wet component enters the room, the temperature and moisture differences gradually increase, and the air temperature and humidity enters the next variable stage.

${{{{{{\rm{CRI}}}}}}}_{\left({{{{{\rm{H}}}}}}\right)}$ and ${{{{{{\rm{CRI}}}}}}}_{\left({{{{{\rm{C}}}}}}\right)}$ both change sharply within t = 0 ~ 6 min, but the maximum value of ${{{{{{\rm{CRI}}}}}}}_{\left({{{{{\rm{H}}}}}}\right)}$ is 1.3 higher and appears 1 min earlier (Fig. 4b). Subsequently, ${{{{{{\rm{CRI}}}}}}}_{\left({{{{{\rm{H}}}}}}\right)}$ increases periodically in a narrow range above 1.0, while ${{{{{{\rm{CRI}}}}}}}_{\left({{{{{\rm{C}}}}}}\right)}$ shows a cyclical decrease.

The dimensionally consistent ${{{{{{\rm{CRI}}}}}}}_{({{{{{\rm{C}}}}}})}^{{{{{{\rm{t}}}}}}}$ and ${{{{{{\rm{CRI}}}}}}}_{({{{{{\rm{H}}}}}})}^{{{{{{\rm{t}}}}}}}$ are obtained by considering the timescale after air temperature and specific humidity are converted into dimensionless ${{{{{{\rm{CRI}}}}}}}_{({{{{{\rm{C}}}}}})}$ and ${{{{{{\rm{CRI}}}}}}}_{({{{{{\rm{H}}}}}})}$. Therefore, ${{{{{{\rm{CRI}}}}}}}_{({{{{{\rm{C}}}}}})}^{{{{{{\rm{t}}}}}}}$ and ${{{{{{\rm{CRI}}}}}}}_{({{{{{\rm{H}}}}}})}^{{{{{{\rm{t}}}}}}}$ can be used to compare the difference of dynamic effects of the same source on the temperature and the humidity environment, which will avail the accurate combination control of air temperature and humidity. As shown in Fig. 4c, ${{{{{{\rm{CRI}}}}}}}_{({{{{{\rm{H}}}}}})}^{{{{{{\rm{t}}}}}}}$ and ${{{{{{\rm{CRI}}}}}}}_{({{{{{\rm{C}}}}}})}^{{{{{{\rm{t}}}}}}}$ first increase rapidly to the maximum value during t = 1 ~ 2 min and t = 1 ~ 3 min respectively, and then both decrease before t = 3 min and t = 4 min. Subsequently, their variation shows similar periodic fluctuation trends. The above phenomena further verify the similarity of the overall trends of sources affecting the air humidity and temperature fields.

In addition, the variation range of ${{{{{{\rm{CRI}}}}}}}_{\left({{{{{\rm{H}}}}}}\right)}$ is much wider than that of ${{{{{{\rm{CRI}}}}}}}_{\left({{{{{\rm{C}}}}}}\right)}$ within the first 5 min, and that of ${{{{{{\rm{CRI}}}}}}}_{({{{{{\rm{H}}}}}})}^{{{{{{\rm{t}}}}}}}$ is also wider than that of ${{{{{{\rm{CRI}}}}}}}_{({{{{{\rm{C}}}}}})}^{{{{{{\rm{t}}}}}}}$, suggesting the initial influence period of the source in the experiment on the humidity environment has the more dynamic characteristics. For the subsequent periodic fluctuations, the fluctuation range of ${{{{{{\rm{CRI}}}}}}}_{({{{{{\rm{H}}}}}})}^{{{{{{\rm{t}}}}}}}$ is narrower than ${{{{{{\rm{CRI}}}}}}}_{({{{{{\rm{C}}}}}})}^{{{{{{\rm{t}}}}}}}$, indicating that the source influencing the air temperature field more notably.

### Source parameters

When source parameters such as the source intensity and source water temperature change, the physical parameters of the wet component will change accordingly, which will affect the heat and moisture exchange between the source and the ambient air. To verify the feasibility of ${{{{{{\rm{CRI}}}}}}}_{({{{{{\rm{H}}}}}})}^{{{{{{\rm{t}}}}}}}$ and ${{{{{{\rm{CRI}}}}}}}_{({{{{{\rm{C}}}}}})}^{{{{{{\rm{t}}}}}}}$ in describing the difference of air temperature and humidity at the same spatial point (X, Y) = (500 mm, 1000 mm) influenced by the source with different source parameters, various moisture source intensity and water temperature conditions are selected for analysis as follows.

**Fig. 5:**
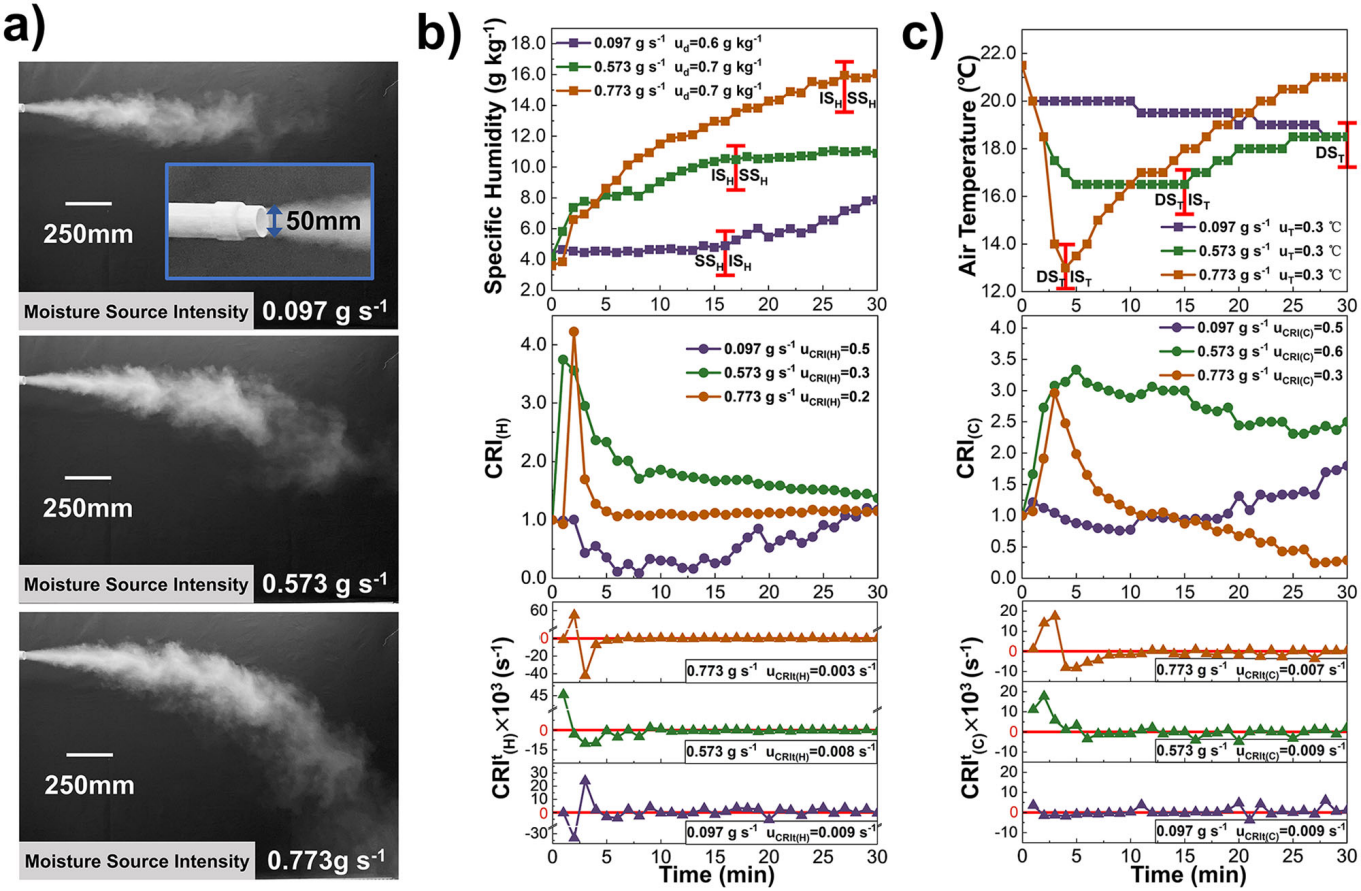
Source Intensity. The variation of Air humidity and temperature, Contribution Ratio of Indoor Humidity (${{{{{{\rm{CRI}}}}}}}_{\left({{{{{\rm{H}}}}}}\right)}$) and Contribution Ratio of Indoor Climate (${{{{{{\rm{CRI}}}}}}}_{\left({{{{{\rm{C}}}}}}\right)}$), Rate of Humidity Contribution Change (${{{{{{\rm{CRI}}}}}}}_{({{{{{\rm{H}}}}}})}^{{{{{{\rm{t}}}}}}}$) and Rate of Indoor Climate Contribution Change (${{{{{{\rm{CRI}}}}}}}_{({{{{{\rm{C}}}}}})}^{{{{{{\rm{t}}}}}}}$) under conditions with various source intensities. **a** Moisture flows produced by the sources with intensities of 0.097 g s^−1^, 0.573 g s^−1^ and 0.773 g s^−1^, respectively. **b** The variation of specific humidity (curves with squares), ${{{{{{\rm{CRI}}}}}}}_{\left({{{{{\rm{H}}}}}}\right)}$ (curves with dots) and ${{{{{{\rm{CRI}}}}}}}_{({{{{{\rm{H}}}}}})}^{{{{{{\rm{t}}}}}}}$ (curves with triangles) at point (X,Y) = (500,1000) mm influenced by the sources with intensities of 0.097 g s^−1^ (purple), 0.573 g s^−1^ (green) and 0.773 g s^−1^ (orange), whose uncertainty is u_d_, u_CRI(H)_ and u_CRIt(H)_ (*n* = 130 samples, 118 samples and 151 samples). **c** The variation of air temperature (curves with squares), ${{{{{{\rm{CRI}}}}}}}_{\left({{{{{\rm{C}}}}}}\right)}$ (curves with dots) and ${{{{{{\rm{CRI}}}}}}}_{({{{{{\rm{C}}}}}})}^{{{{{{\rm{t}}}}}}}$ (curves with triangles) at point (X,Y) = (500, 1000) mm influenced by the sources with intensities of 0.097 g s^-1^ (purple), 0.573 g s^-1^ (green) and 0.773 g s^-1^ (orange), whose uncertainty is u_T_, u_CRI(C)_ and u_CRIt(C)_ (*n* = 130 samples, 118 samples and 151 samples).

Figure 5a shows the moisture flows produced by the sources with intensities of 0.097 g s^−1^, 0.573 g s^−1^ and 0.773 g s^−1^. As shown in Fig. 5b, moisture sources of different intensities all contribute to an increase in air humidity over time, but their exact variation tendencies of specific humidity, ${{{{{{\rm{CRI}}}}}}}_{\left({{{{{\rm{H}}}}}}\right)}$ and ${{{{{{\rm{CRI}}}}}}}_{({{{{{\rm{H}}}}}})}^{{{{{{\rm{t}}}}}}}$ differ from each other. When the source intensity is 0.097 g s^−1^, the specific humidity does not increase distinctly until t = 16 min. The humidity of source intensity 0.573 g s^−1^ and 0.773 g s^−1^ undergoes the increase stage followed by the stable stage, while the latter one has the longer increase stage with a larger growth rate, indicating that a higher intensity source has a more notable effect on improving indoor humidity environment. When the source intensity is relatively large, the moisture rapidly diffuses into the whole space, which result in a small difference between the increase in humidity at this point and the average increase in humidity throughout the space. Therefore, the variation of ${{{{{{\rm{CRI}}}}}}}_{\left({{{{{\rm{H}}}}}}\right)}$ under source intensity 0.773 g s^−1^ is basically stable at 1.0 after t = 5 min.

The ${{{{{{\rm{CRI}}}}}}}_{({{{{{\rm{H}}}}}})}^{{{{{{\rm{t}}}}}}}$ of each source intensity condition mainly fluctuates around 0 after the dramatic change. Under the source intensity 0.773 g s^−1^, ${{{{{{\rm{CRI}}}}}}}_{({{{{{\rm{H}}}}}})}^{{{{{{\rm{t}}}}}}}$ reaches 0.055 s^−1^ at t = 2 min and -0.042 s^−1^ at t = 3 min. Subsequently, the humidity difference between the air and the source declines, the evaporation rate reduces, and ${{{{{{\rm{CRI}}}}}}}_{({{{{{\rm{H}}}}}})}^{{{{{{\rm{t}}}}}}}$ begins to fluctuate. When the intensity is 0.573 g s^−1^, ${{{{{{\rm{CRI}}}}}}}_{({{{{{\rm{H}}}}}})}^{{{{{{\rm{t}}}}}}}$ fluctuates widely in the range of -0.010 ~ 0.002 s^−1^ during the t = 2 ~ 9 min and then fluctuates based on ${{{{{{\rm{CRI}}}}}}}_{({{{{{\rm{H}}}}}})}^{{{{{{\rm{t}}}}}}}$ = 0. The ${{{{{{\rm{CRI}}}}}}}_{({{{{{\rm{H}}}}}})}^{{{{{{\rm{t}}}}}}}$ of 0.097 g s^−1^ also fluctuates around 0 after peaking at 0.024 s^−1^, but has a wider fluctuation range than that of 0.573 g s^−1^ and 0.773 g s^−1^.

In Fig. 5c, the air temperature decreases continuously for a source intensity of 0.097 g s^−1^, while it starts to rise after decreasing to a minimum in the other conditions. Correspondingly, the ${{{{{{\rm{CRI}}}}}}}_{\left({{{{{\rm{C}}}}}}\right)}$ of 0.097 g s^−1^ increases in general but it of 0.573 g s^−1^ and 0.773 g s^−1^ decreases after increasing to the maximum value 3.3 and 3.0, respectively. As the source intensity increases, the air temperature decreases at a shorter DS_T_. The air temperature of source intensity 0.773 g s^−1^ decreases by 8.5 °C with only 4 min. The reason may be that a larger intensity source produces more wet-component per second, and the latent and sensible heat exchange between it and the surrounding air are enhanced.

The ${{{{{{\rm{CRI}}}}}}}_{({{{{{\rm{C}}}}}})}^{{{{{{\rm{t}}}}}}}$ variation is different among various source intensity conditions. It fluctuates slightly around 0 at 0.097 g s^−1^. When the source intensity is 0.573 g s^−1^, the ${{{{{{\rm{CRI}}}}}}}_{({{{{{\rm{C}}}}}})}^{{{{{{\rm{t}}}}}}}$ increases from 0.011 s^−1^ to 0.018 s^−1^ and decreases continuously within t = 2 ~ 6 min, then fluctuates periodically at the range of -0.005 ~ 0.002 s^−1^. Under the intensity condition of 0.773 g s^−1^, ${{{{{{\rm{CRI}}}}}}}_{({{{{{\rm{C}}}}}})}^{{{{{{\rm{t}}}}}}}$ varies remarkably during the first 8 min, and fluctuates periodically with the smaller range than the conditions of 0.097 g s^−1^ and 0.573 g s^−1^. Thus, as the source intensity increases, the dynamic effect of the source on the indoor thermal environment becomes more notable during the DS_T_ but less prominent during the IS_T_.

**Fig. 6:**
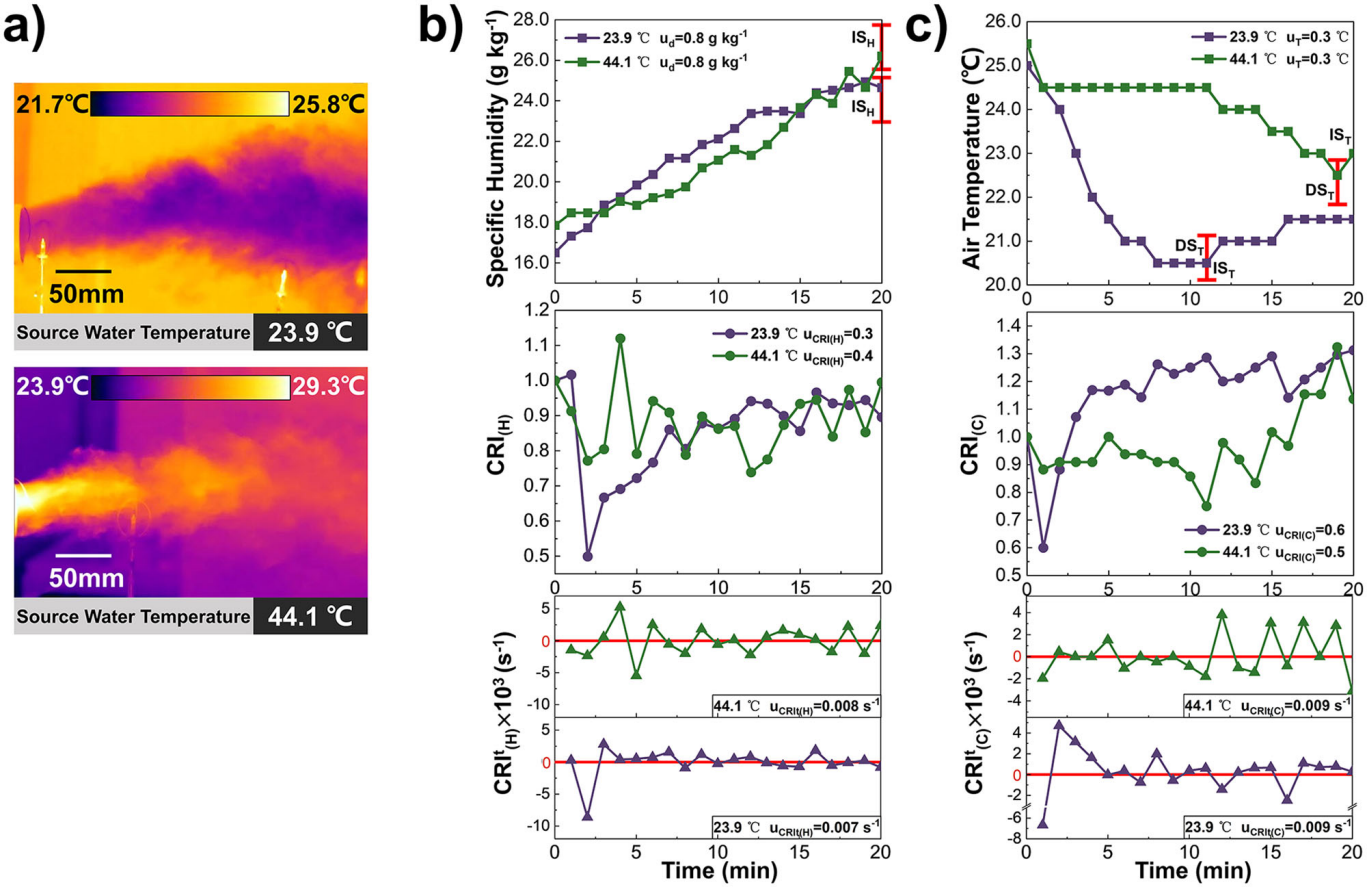
Source Water Temperature. The variation of Air humidity and temperature, Contribution Ratio of Indoor Humidity (${{{{{{\rm{CRI}}}}}}}_{\left({{{{{\rm{H}}}}}}\right)}$) and Contribution Ratio of Indoor Climate (${{{{{{\rm{CRI}}}}}}}_{\left({{{{{\rm{C}}}}}}\right)}$), Rate of Humidity Contribution Change (${{{{{{\rm{CRI}}}}}}}_{({{{{{\rm{H}}}}}})}^{{{{{{\rm{t}}}}}}}$) and Rate of Indoor Climate Contribution Change (${{{{{{\rm{CRI}}}}}}}_{({{{{{\rm{C}}}}}})}^{{{{{{\rm{t}}}}}}}$) under conditions with various source water temperatures. **a** Images of moisture flows produced by the sources with water temperatures of 23.9 °C and 44.1 °C taken by the FLIR T500 infrared thermal imager. **b** The variation of specific humidity (curves with squares), ${{{{{{\rm{CRI}}}}}}}_{\left({{{{{\rm{H}}}}}}\right)}$ (curves with dots) and ${{{{{{\rm{CRI}}}}}}}_{({{{{{\rm{H}}}}}})}^{{{{{{\rm{t}}}}}}}$ (curves with triangles) at point (X,Y) = (500,1000) mm influenced by the sources with water temperatures of 23.9 °C (purple) and 44.1 °C (green), whose uncertainty is u_d_, u_CRI(H)_ and u_CRIt(H)_ (*n* = 135 samples and 142 samples). **c** The variation of air temperature (curves with squares), ${{{{{{\rm{CRI}}}}}}}_{\left({{{{{\rm{C}}}}}}\right)}$ (curves with dots) and ${{{{{{\rm{CRI}}}}}}}_{({{{{{\rm{C}}}}}})}^{{{{{{\rm{t}}}}}}}$ (curves with triangles) at point (X,Y) = (500,1000) mm influenced by the sources with water temperatures of 23.9 °C (purple) and 44.1 °C (green), whose uncertainty is u_T_, u_CRI(C)_ and u_CRIt(C)_ (*n* = 135 samples and 142 samples).

Figure 6a shows the infrared thermal Images of moisture flows produced by the sources with water temperatures of 23.9 °C and 44.1 °C. The variation of air specific humidity and temperature, ${{{{{{\rm{CRI}}}}}}}_{\left({{{{{\rm{H}}}}}}\right)}$ and ${{{{{{\rm{CRI}}}}}}}_{\left({{{{{\rm{C}}}}}}\right)}$, ${{{{{{\rm{CRI}}}}}}}_{({{{{{\rm{H}}}}}})}^{{{{{{\rm{t}}}}}}}$ and ${{{{{{\rm{CRI}}}}}}}_{({{{{{\rm{C}}}}}})}^{{{{{{\rm{t}}}}}}}$ under different source water temperature conditions are shown in Fig. 6b and Fig. 6c. The specific humidity increases continuously for both source water temperature conditions. Under the water temperature condition of 44.1 °C and 23.9 °C, the specific humidity increases by 8.2 g kg^−1^ and 8.4 g kg^−1^ within 20 min, respectively. Concurrently, the ${{{{{{\rm{CRI}}}}}}}_{\left({{{{{\rm{H}}}}}}\right)}$ of 23.9 °C decreases and then increases while that of 44.1 °C fluctuates between 0.7 and 1.1 before the source is switched off. As the water temperature decreases from 44.1 °C to 23.9 °C, the fluctuation range of ${{{{{{\rm{CRI}}}}}}}_{({{{{{\rm{H}}}}}})}^{{{{{{\rm{t}}}}}}}$ during t = 8 ~ 30 min shrinks from −0.002 ~ 0.002 s^−1^ to -0.001 ~ 0.002 s^−1^. This means that a source with a higher water temperature has a more dynamical impact on the indoor humidity field.

As shown in Fig. 6c, the air temperature first decreases and then increases with time for both water temperature conditions. However, when the source water temperature is 44.1 °C, the decrease stage of air temperature does not begin until t = 11 min, but its duration is 3 min less than that of the source water temperature 23.9 °C. The main reason is that the amount of sensible heat transferred from the high-temperature wet component to the air is comparable to the latent heat transferred from the air due to moisture evaporation, which results in air temperature remaining constant for the first 11 min. The latent heat exchange rate at this point becomes larger than the sensible one as the wet-component evaporation rate increases continuously, and the air temperature drops. As the humidity difference between the source and the air decreases, the stronger sensible heat exchange of the high-water temperature condition quickly takes over the dominant role of the latent one, and the duration of DS_T_ at the 44.1°C condition is shorter. Besides, the remarkable change period of ${{{{{{\rm{CRI}}}}}}}_{({{{{{\rm{C}}}}}})}^{{{{{{\rm{t}}}}}}}$ appears at t = 0 ~ 5 min when the water temperature is 23.9 °C, and that of water temperature condition 44.1 °C appears at the last 11 min, which suggests that the significant impact stage of source with higher water temperature on the air temperature distribution appears later.

Overall, when the source intensity is constant, an appropriate increase in the source water temperature can improve the indoor humidity, while reducing its effect on the indoor air temperature field as a negative heat source.

Furthermore, as for sources of different source parameters influencing the same indoor environment, if the ${{{{{\rm{CRI}}}}}}$ influenced by each source increases or decreases by the same value, the difference in influence speed and efficiency of each studied source on the indoor humidity field or temperature field can be determined more accurately, according to the variation range and trend of ${{{{{{\rm{CRI}}}}}}}^{{{{{{\rm{t}}}}}}}$. This is conducive to the subsequent selection of heat and moisture source parameters in a variety of indoor environment dynamic control situations.

### Indoor environmental conditions

The indoor environment with different air temperatures and humidity will modify the temperature and moisture differences between the source and ambient air, which will affect the effect of the source on the indoor temperature and humidity distribution. Moreover, the rates of heat transfer, evaporation, and diffusion are also different for various atmospheric pressures. As follows, the impact of the humidifier as a moisture and heat source on the indoor environment with different ambient temperatures, humidity and atmospheric pressure is analyzed by using the presently proposed indexes ${{{{{{\rm{CRI}}}}}}}_{({{{{{\rm{C}}}}}})}^{{{{{{\rm{t}}}}}}}$ and ${{{{{{\rm{CRI}}}}}}}_{({{{{{\rm{H}}}}}})}^{{{{{{\rm{t}}}}}}}$.

**Fig. 7:**
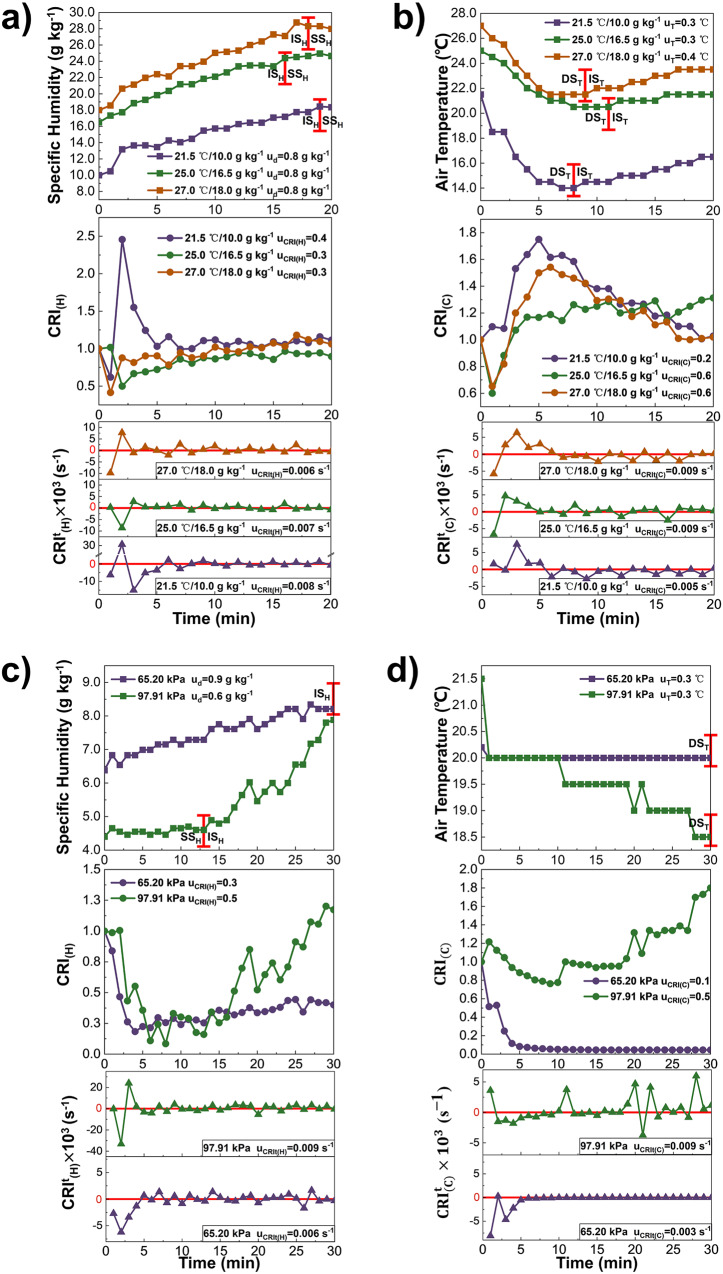
Ambient Temperature, Humidity and Pressure. The variation of Air humidity and temperature, Contribution Ratio of Indoor Humidity (${{{{{{\rm{CRI}}}}}}}_{\left({{{{{\rm{H}}}}}}\right)}$) and Contribution Ratio of Indoor Climate (${{{{{{\rm{CRI}}}}}}}_{\left({{{{{\rm{C}}}}}}\right)}$), Rate of Humidity Contribution Change (${{{{{{\rm{CRI}}}}}}}_{({{{{{\rm{H}}}}}})}^{{{{{{\rm{t}}}}}}}$) and Rate of Indoor Climate Contribution Change (${{{{{{\rm{CRI}}}}}}}_{({{{{{\rm{C}}}}}})}^{{{{{{\rm{t}}}}}}}$) under various environmental conditions. **a** The variation of specific humidity (curves with squares), ${{{{{{\rm{CRI}}}}}}}_{\left({{{{{\rm{H}}}}}}\right)}$ (curves with dots) and ${{{{{{\rm{CRI}}}}}}}_{({{{{{\rm{H}}}}}})}^{{{{{{\rm{t}}}}}}}$ (curves with triangles) at point (X,Y) = (500,1000) mm influenced by the sources under various ambient air temperatures and humidities (21^.^5 °C/10.0 g kg^-1^), (25.0 °C/16.5 g kg^-1^) and (27.0 °C/18.0 g kg^-1^), whose uncertainty is u_d_, u_CRI(H)_ and u_CRIt(H)_ (*n* = 123 samples, 135 samples and 108 samples). **b** The variation of air temperature (curves with squares), ${{{{{{\rm{CRI}}}}}}}_{\left({{{{{\rm{C}}}}}}\right)}$ (curves with dots) and ${{{{{{\rm{CRI}}}}}}}_{({{{{{\rm{C}}}}}})}^{{{{{{\rm{t}}}}}}}$ (curves with triangles) at point (X,Y) = (500, 1000) mm influenced by the sources under various ambient air temperatures and humidities (21.5 °C/10.0 g kg^−1^), (25.0 °C/16.5 g kg^−1^) and (27.0 °C/18.0 g kg^−1^), whose uncertainty is u_T_, u_CRI(C)_ and u_CRIt(C)_ (*n* = 123 samples, 135 samples and 108 samples). **c** The variation of specific humidity (curves with squares), ${{{{{{\rm{CRI}}}}}}}_{\left({{{{{\rm{H}}}}}}\right)}$ (curves with dots) and ${{{{{{\rm{CRI}}}}}}}_{({{{{{\rm{H}}}}}})}^{{{{{{\rm{t}}}}}}}$ (curves with triangles) at point (X,Y) = (500, 1000) mm influenced by the sources under atmospheric pressures 65.20 kPa (purple) and 97.91 kPa (green), whose uncertainty is u_d_, u_CRI(H)_ and u_CRIt(H)_ (*n* = 135 samples and 130 samples). **d** The variation of air temperature (curves with squares), ${{{{{{\rm{CRI}}}}}}}_{\left({{{{{\rm{C}}}}}}\right)}$ (curves with dots) and ${{{{{{\rm{CRI}}}}}}}_{({{{{{\rm{C}}}}}})}^{{{{{{\rm{t}}}}}}}$ (curves with triangles) at point (X,Y) = (500,1000) mm influenced by the sources under atmospheric pressures 65.20 kPa (purple) and 97.91 kPa (green), whose uncertainty is u_T_, u_CRI(C)_ and u_CRIt(C)_ (*n* = 135 samples and 130 samples).

When the ambient air temperature and specific humidity are (21.5 °C, 10.0 g kg^−1^), (25.0 °C, 16.5 g kg^−1^) and (27.0 °C, 18.0 g kg^−1^), the indices variation at the same spatial point (X, Y) = (500 mm, 1000 mm) is shown in Fig. 7a and Fig. 7b. The specific humidity for these conditions increases by 8.4 g kg^−1^, 8.2 g kg^−1^, and 10.0 g kg^−1^ in turn before the source is turned off. When the ambient temperature and humidity is 21.5 °C and 10.0 g kg^−1^ respectively, the maximum ${{{{{{\rm{CRI}}}}}}}_{({{{{{\rm{H}}}}}})}^{{{{{{\rm{t}}}}}}}$ is as high as 0.031 s^−1^. The probable reason is that the moisture difference between the source and the indoor air is much larger due to the smaller initial ambient humidity 10.0 g kg^−1^, and the droplet evaporation is accelerated.

In Fig. 7b, the air temperature of each condition rapidly decreases and then increases gradually, and the transition from DS_T_ to IS_T_, where the lowest temperature appears, is in the range of t = 8 ~ 11 min. When the ambient air temperature is 21.5 °C, 25.0 °C and 27.0 °C, ${{{{{{\rm{CRI}}}}}}}_{\left({{{{{\rm{C}}}}}}\right)}$ increases by 0.6, 0.3 and 0.4 during the DS_T_. It of 21.5 °C and 27.0 °C respectively decreases by 0.6 and 0.4 during the IS_T_, but that of 25.0 °C remains stable around 1.3. The ${{{{{{\rm{CRI}}}}}}}_{({{{{{\rm{C}}}}}})}^{{{{{{\rm{t}}}}}}}$ decreases or increases notably at t = 0 ~ 5 min, then shows a fluctuating trend. The maximum ${{{{{{\rm{CRI}}}}}}}_{({{{{{\rm{C}}}}}})}^{{{{{{\rm{t}}}}}}}$ of ambient temperature condition 21.5 °C, 25.0 °C and 27.0 °C is 0.007 s^−1^, 0.005 s^−1^ and 0.006 s^−1^, respectively. The reason can be that, during the experimental test, the source water temperature fluctuated and the temperature difference between source and indoor air became 6.6 °C, -0.3 °C and 0.1 °C accordingly. As this temperature difference increases, the intensity of the heat exchange between the source and the air becomes more pronounced.

In summary, when the source water temperature is higher than the ambient temperature, lowering the ambient temperature can increase the temperature difference between them and accelerate the heat exchange between the wet component and the air. In addition, the difference in humidity between ambient air and the source remains the crucial impact factor for the source influencing the indoor humidity environment.

As shown in Fig. 7c, the trends of the air humidity variation at 65.20 kPa and 97.91 kPa are clearly different from each other. At an atmospheric pressure of 65.20 kPa, the specific humidity increases slightly by 1.7 g kg^−1^ within 30 min. At 97.91 kPa, specific humidity eventually increases by 3.4 g kg^−1^ and ${{{{{{\rm{CRI}}}}}}}_{\left({{{{{\rm{H}}}}}}\right)}$ increases to 1.2, with the small water vapor diffusion coefficient. Nevertheless, when the source is turned on for 30 min, ${{{{{{\rm{CRI}}}}}}}_{\left({{{{{\rm{H}}}}}}\right)}$ of 65.20 kPa is as low as 0.5. The reason can be that when the wet-component reaches a spatial point, it evaporates into the water vapor due to the moisture difference, and the air humidity briefly rises. Subsequently, due to the large water vapor diffusion coefficient ${D}_{i,m}$ at low pressure, according to the heat and mass exchange equations between the droplets and air (Eqs. 1–3)^35–37^, the majority of water vapor stays at this location only for a short time and rapidly diffuses into the peri-bacteroid space, so that the specific humidity at this point is reduced. As a result, the source has less effect on improving the indoor humidity environment at low pressure. The remarkable variation ranges of ${{{{{{\rm{CRI}}}}}}}_{({{{{{\rm{H}}}}}})}^{{{{{{\rm{t}}}}}}}$ at 97.91 kPa and 65.20 kPa appear within the first 5 min, and it of both atmospheric pressure conditions fluctuates periodically with the baseline ${{{{{{\rm{CRI}}}}}}}_{({{{{{\rm{H}}}}}})}^{{{{{{\rm{t}}}}}}}$ = 0 after that.

1 $$\frac{d{m}_{{{{{{\rm{p}}}}}}}}{{dt}}={k}_{{{{{{\rm{c}}}}}}}{A}_{{{{{{\rm{p}}}}}}}\rho {{{{{\rm{ln}}}}}}\left(1+{B}_{{{{{{\rm{m}}}}}}}\right)=\frac{{D}_{{{{{{\rm{i}}}}}},{{{{{\rm{m}}}}}}}Sh}{{d}_{{{{{{\rm{p}}}}}}}}{A}_{{{{{{\rm{p}}}}}}}\rho {{{{{\rm{ln}}}}}}\left(1+{B}_{{{{{{\rm{m}}}}}}}\right)$$

2 $${D}_{{{{{{\rm{i}}}}}},{{{{{\rm{m}}}}}}}={D}_{0}\frac{{p}_{0}}{p}{\left(\frac{T}{{T}_{0}}\right)}^{\frac{3}{2}}$$

3 $${m}_{{{{{{\rm{p}}}}}}}{c}_{{{{{{\rm{p}}}}}}}\frac{d{T}_{{{{{{\rm{p}}}}}}}}{{dt}}=h{A}_{{{{{{\rm{p}}}}}}}\left({T}_{{{\infty }}}-{T}_{{{{{{\rm{p}}}}}}}\right)-\frac{d{m}_{{{{{{\rm{p}}}}}}}}{{dt}}{h}_{{{{{{\rm{fg}}}}}}}+{\varepsilon }_{{{{{{\rm{p}}}}}}}{A}_{{{{{{\rm{p}}}}}}}\sigma ({\theta }_{{{{{{\rm{R}}}}}}}^{4}-{T}_{{{{{{\rm{p}}}}}}}^{4})$$

where ${m}_{{{{{{\rm{p}}}}}}}$ is the mass of droplet (kg), ${k}_{{{{{{\rm{c}}}}}}}$ is the mass transfer coefficient (m s^−1^), ${A}_{{{{{{\rm{p}}}}}}}$ is the surface area of droplet (m^2^), $\rho$ is the air density (kg m^-3^), ${B}_{{{{{{\rm{m}}}}}}}$ is the Spalding mass number, ${D}_{{{{{{\rm{i}}}}}},{{{{{\rm{m}}}}}}}$ is the diffusion coefficient of water vapor in the air at pressure $p$ and temperature $T$ (m^2^ s^−1^), $Sh$ is the Sherwood number, ${d}_{{{{{{\rm{p}}}}}}}$ is the droplet size (m), ${D}_{0}$ is the diffusion coefficient of water vapor in the air when ${p}_{0}$ = 101.30 kPa and ${T}_{0}$ = 273.0 K, which is taken as 0.22 × 10^-4^ m^2^ s^−1^, ${c}_{{{{{{\rm{p}}}}}}}$ is the specific heat capacity of droplet at constant pressure (J kg^−1^ K^−1^), ${T}_{{{{{{\rm{p}}}}}}}$ is the temperature of droplet (K), $h$ is the convective heat transfer coefficient (W m^-2^ K^−1^), ${T}_{\infty }$ is the ambient air temperature (K), ${h}_{{{{{{\rm{fg}}}}}}}$ is the amount of latent heat exchange (J kg^−1^), ${\varepsilon }_{{{{{{\rm{p}}}}}}}$ is the droplet emissivity, $\sigma$ is Stefan-Boltzmann constant, 5.67 × 10^-8^ (W m^-2^ K^-4^), ${\theta }_{{{{{{\rm{R}}}}}}}$ is the radiation temperature (K).

In Fig. 7d, the source used in atmospheric pressure conditions of 65.20 kPa and 97.91 kPa have a small source intensity and elevated source water temperature, so the air temperature remains constant or then decreases after that. Under the condition of 65.20 kPa, ${{{{{{\rm{CRI}}}}}}}_{({{{{{\rm{C}}}}}})}^{{{{{{\rm{t}}}}}}}$ barely change during most of the time when the source is operating.

## Conclusions

The variation of air humidity under the influence of a moisture source can be divided into an increase stage and a stable stage, while that of air temperature under the influence of it as a heat source can be divided into a decrease stage and an increase stage. The dynamic characteristics of the source influencing on the indoor humidity and thermal environment in each stage can be portrayed more intuitively and accurately by the proposed indexes ${{{{{{\rm{CRI}}}}}}}_{({{{{{\rm{H}}}}}})}^{{{{{{\rm{t}}}}}}}$ and ${{{{{{\rm{CRI}}}}}}}_{({{{{{\rm{C}}}}}})}^{{{{{{\rm{t}}}}}}}$, which is conducive to further detailed transient control of the indoor environment.

Based on the variation of ${{{{{{\rm{CRI}}}}}}}_{({{{{{\rm{H}}}}}})}^{{{{{{\rm{t}}}}}}}$ and ${{{{{{\rm{CRI}}}}}}}_{({{{{{\rm{C}}}}}})}^{{{{{{\rm{t}}}}}}}$, we found that the dynamical influence of the source on the indoor environment is quite different for various source parameters and environmental parameters. When the source water temperature is higher than the ambient temperature, increasing the source intensity and source water temperature can appropriately raise the air humidity while reducing the effect of the moisture source as a negative heat source on the air temperature. When the intensity and water temperature are fixed, the influence of the source on the cryogenic drying environment becomes more notable. In addition, the source used in this study has less effect on the improvement of indoor humidity levels at low pressure.

## Methods

### Moisture source and heat source

**Fig. 8:**
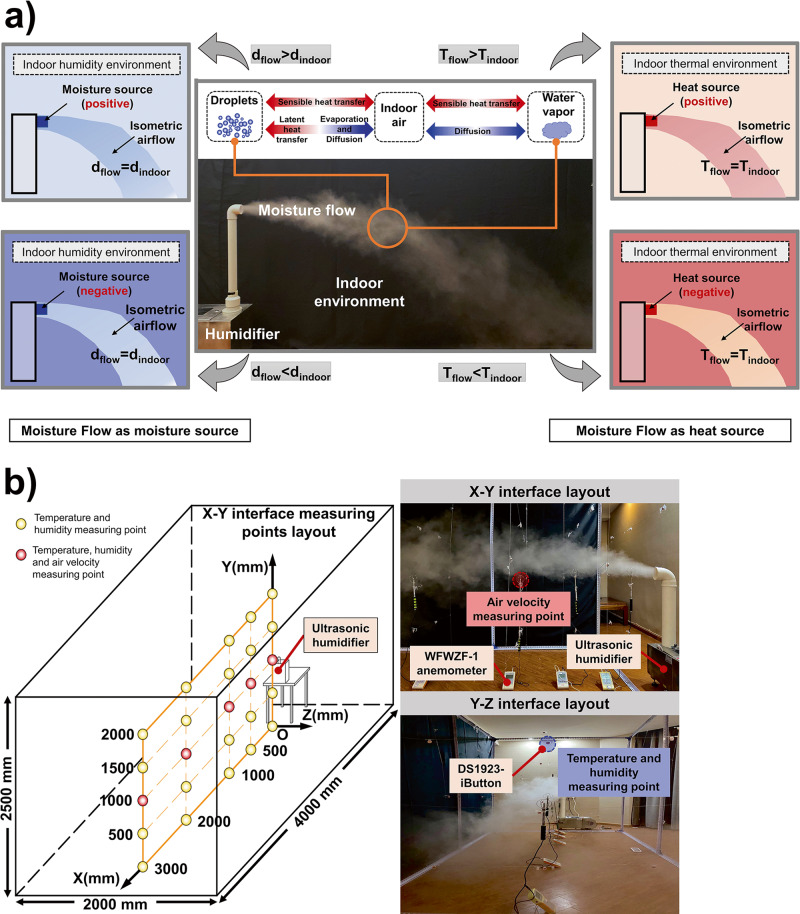
Feasibility Verification Experiment. Feasibility verification experiment of Rate of Humidity Contribution Change (${{{{{{\rm{CRI}}}}}}}_{({{{{{\rm{H}}}}}})}^{{{{{{\rm{t}}}}}}}$) and Rate of Indoor Climate Contribution Change (${{{{{{\rm{CRI}}}}}}}_{({{{{{\rm{C}}}}}})}^{{{{{{\rm{t}}}}}}}$) by an ultrasonic humidifier under various source parameters and environmental conditions. **a** Moisture flow as a moisture and heat source. When a humidifier is used to deliver moisture to a room, the airflow (air humidity d_flow_, air temperature T_flow_) generated by the device exchanges the moisture and heat with the indoor air (air humidity d_indoor_, air temperature T_indoor_) simultaneously, which can be regarded as a moisture source for the humidity field and a heat source for the temperature field. **b** Layout of the measuring points and measurement facilities. The air temperature and humidity measuring points are arranged in the XOY plane (X = 0, 500, 1000, 2000, 3000 mm, Y = 0, 500, 1000, 1500 and 2000 mm), and the air velocity measuring points are arranged at X = 0, 500, 1000, 2000 and 3000 mm along the outlet axis of the source. The sampling interval is set to 1 min.

To facilitate the analysis of the moisture flow which is delivered by the humidifier, affecting the indoor humidity field, it can be considered as two parts, the airflow with humidity equal to the ambient humidity and the moisture source located at the humidifier outlet (Fig. 8a). If the moisture flow has a greater humidity than the ambient air, the wet component diffuses continuously and raises the humidity level of the indoor environment. It is considered as a positive moisture source and an iso-humid flow. Conversely, when a flow with fewer components than the ambient air enters the room, it can be considered as a negative moisture source and an isometric flow.

On the other hand, when analyzing the influence of the humidity airflow on the temperature field, it can be considered as a heat source and the flow with a temperature equals the initial indoor temperature^30^. The heat exchange between the heat source and the isothermal flow consists of sensible heat transfer and latent heat transfer due to droplet evaporation. If the airflow is heated by the heat source and then transfers heat to the indoor air in general, resulting in an increase of the air temperature, it can be considered as a positive heat source and an isothermal flow, and vice versa as a negative heat source and the isometric flow.

### Experimental procedure

The specific arrangement of the experiment is shown in Fig. 8b. Firstly, the air conditioning system runs for 180 minutes to ensure that the temperature and humidity in the experimental space (4000 mm (length) × 2000 mm (width) × 2500 mm (height)) meet the requirements of operating conditions and remain stable. Subsequently, the outlet of the moisture source was placed at 1000 mm height (i.e. 1/2 of the height of the experimental space). The moisture source was turned on after the air-conditioner was switched off, allowing the moisture air to flow into the test chamber at a certain speed, and was closed after 30 minutes of steady operation. To prevent the current experiment from influencing the next one, the air conditioning and a dehumidifier were used to bring the ambient temperature and humidity up to the requirements of the next experimental conditions. The test parameters include air temperature, relative humidity, air velocity, atmospheric pressure, and the temperature and mass of water in the moisture source water tank. The air temperature is used to calculate ${{{{{{\rm{CRI}}}}}}}_{\left({{{{{\rm{C}}}}}}\right)}$ and ${{{{{{\rm{CRI}}}}}}}_{({{{{{\rm{C}}}}}})}^{{{{{{\rm{t}}}}}}}$. The specific humidity, calculated from the air temperature, relative humidity and atmospheric pressure, is used to calculate ${{{{{{\rm{CRI}}}}}}}_{\left({{{{{\rm{H}}}}}}\right)}$ and ${{{{{{\rm{CRI}}}}}}}_{({{{{{\rm{H}}}}}})}^{{{{{{\rm{t}}}}}}}$. And the mass of water in the tank before and after the experiment is used to calculate the humidification capacity (namely the source intensity).

### Measurement facilities

The air temperature and relative humidity at each measurement point were recorded using the DS1923-iButton. The measurement ranges of this instrument are −10.0 ~ 50.0 °C and 10 ~ 100 %RH, and the measurement accuracy is ±0.5 °C and ±5 %RH, respectively. The air velocity was measured by a WFWZF−1 anemometer with a measuring range and accuracy of 0.05 ~ 3.00 m s^−1^ and ±0.05 m s^−1^. The temperature of water in the tank was measured by an HT−1420K thermocouple with a measuring range of -50.0 ~ 1600.0 °C and an accuracy of ±0.75%t. The ambient atmospheric pressure was measured by the SSN-71 barometer with a measurement range of 30.00 to 110.00 kPa and an accuracy of 0.01 kPa. The measuring range and accuracy of all experimental instruments are in line with the related requirements of “Evaluation standard for indoor thermal environment in civil buildings” (GB/T 50785-2012)^38^.

### Linear characteristic of humidity and temperature field

When forced convection is the dominant form of airflow affecting the indoor environment, the distribution of the indoor humidity and temperature fields, influenced by its function as a moisture and heat source, has a linear characteristic^28^. For the humidity field, since the specific humidity is a passive scalar, the effects of vapor diffusion and droplet evaporation can be analyzed separately when the position and intensity of the moisture source are fixed. The variation of the humidity under the influence of the source is a linear superposition of the humidification effects of the two processes.

4 $$\rho V\frac{d\bar{X}}{{dt}}={\rho }_{{{{{{\rm{f}}}}}}}{V}_{{{{{{\rm{f}}}}}}}({X}_{{{{{{\rm{vapor}}}}}}}+{X}_{{{{{{\rm{droplet}}}}}}})$$

where $\rho$ is the air density of the indoor air (kg m^-3^), $V$ is the conditioned room volume (m^3^), $\bar{X}$ is the average indoor specific humidity of the room (g kg^−1^), $t$ is the time (s), ${\rho }_{{{{{{\rm{f}}}}}}}$ is the air density of the humidity flow (kg m^-3^); ${V}_{{{{{{\rm{f}}}}}}}$ is the air flow rate of the humidity airflow (m^3^ s^−1^), ${X}_{{{{{{\rm{vapor}}}}}}}$ and ${X}_{{{{{{\rm{droplet}}}}}}}$ is the variation of specific humidity influenced by the water vapor diffusion and droplet evaporation, respectively (g kg^−1^).

Similarly, the air temperature is a scalar quantity. When the intensity and location of a heat source remain unchanged or change little, the effect of the source on the temperature distribution can be viewed as a superposition of sensible heat exchange between water vapor or droplets and the indoor air, and latent heat exchange between droplets and air.

5 $$\frac{\partial \theta }{\partial t}+{{{{{{\bf{u}}}}}}}_{{{{{{\bf{i}}}}}}}\cdot \frac{\partial \theta }{\partial {x}_{i}}=\frac{\partial }{\partial {x}_{i}}\left(\frac{{\nu }_{t}}{{\Pr }_{t}}\cdot \frac{\partial \theta }{\partial {x}_{i}}\right)+\frac{{q}_{{{{{{\rm{sensible}}}}}}}}{{c}_{p}\cdot \rho }+\frac{{q}_{{{{{{\rm{latent}}}}}}}}{{c}_{p}\cdot \rho }$$

where $\theta$ is the air temperature (°C), ${{{{{{\bf{u}}}}}}}_{{{{{{\bf{i}}}}}}}$ is the air velocity (m s^−1^), including ${{{{{{\bf{u}}}}}}}_{{{{{{\bf{x}}}}}}}$, ${{{{{{\bf{u}}}}}}}_{{{{{{\bf{y}}}}}}}$ and ${{{{{{\bf{u}}}}}}}_{{{{{{\bf{z}}}}}}}$, ${x}_{i}$ is the component of the spatial coordinates, including ${x}_{x}$, ${x}_{y}$ and ${x}_{z}$, ${\nu }_{t}$ is the air turbulent viscosity (kg m^-1^ s^−1^), ${\Pr }_{t}$ is the turbulent Prandtl number, ${c}_{p}$ is the specific heat of indoor air (J kg^−1^ K^-1^), ${q}_{{{{{{\rm{sensible}}}}}}}$ and ${q}_{{{{{{\rm{latent}}}}}}}$ is the sensible and latent heat transfer rate between humidity airflow and indoor air, respectively (W).

### From ${{{{{{\bf{CRI}}}}}}}_{({{{{{\bf{H}}}}}})}$ to ${{{{{{\bf{CRI}}}}}}}_{({{{{{\bf{H}}}}}})}^{{{{{{\bf{t}}}}}}}$

**Fig. 9:**
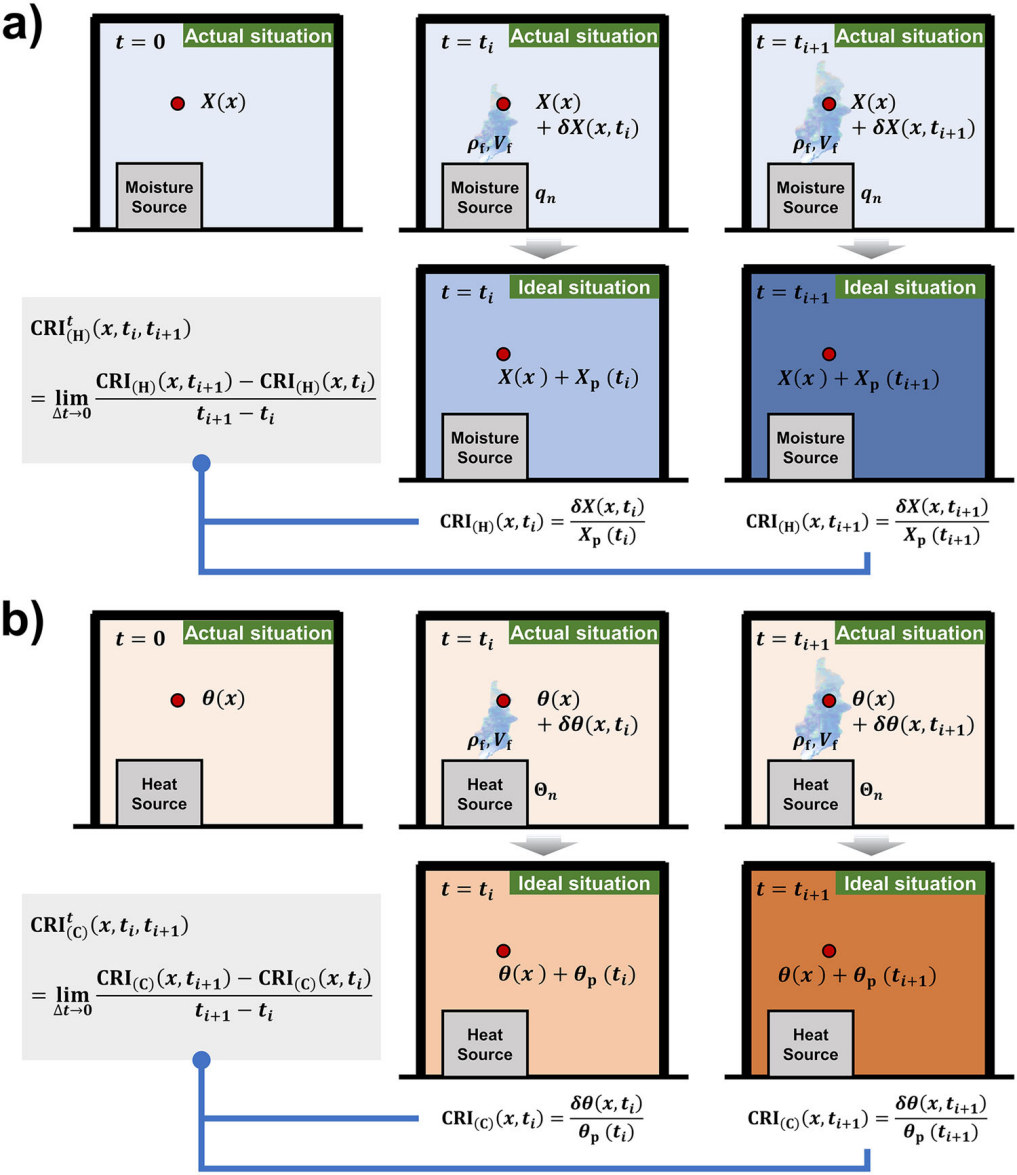
Indices development. **a** From Contribution Ratio of Indoor Humidity (${{{{{{\rm{CRI}}}}}}}_{\left({{{{{\rm{H}}}}}}\right)}$) to Rate of Humidity Contribution Change ${{{{{{\rm{CRI}}}}}}}_{({{{{{\rm{H}}}}}})}^{{{{{{\rm{t}}}}}}}$ with considering the dynamic variation per unit time. The humidity at a spatial point $x$ is $X(x)$ at $t=0$. After the moisture source with source intensity ${q}_{n}$ delivers the humidity flow (air density ${\rho }_{{{{{{\rm{f}}}}}}}$, air flow rate ${V}_{{{{{{\rm{f}}}}}}}$) to the indoor environment, the humidity at point $x$ varies by $\delta X(x,{t}_{i})$ at $t={t}_{i}$ and $\delta X(x,{t}_{i+1})$ at $t={t}_{i+1}$ under actual situation, which varies by ${X}_{{{{{{\rm{p}}}}}}}({t}_{i})$ at $t={t}_{i}$ and ${X}_{{{{{{\rm{p}}}}}}}({t}_{i+1})$ at $t={t}_{i+1}$ under ideal situation (i.e. perfect mixing condition). The ratio of $\delta X(x,{t}_{i})$ to ${X}_{{{{{{\rm{p}}}}}}}({t}_{i})$ is the Contribution Ratio of Indoor Humidity at point $x$ and time ${t}_{i}$ (${{{{{{\rm{CRI}}}}}}}_{\left({{{{{\rm{H}}}}}}\right)}(x,{t}_{i})$), and the ratio of $\delta X(x,{t}_{i+1})$ to ${X}_{{{{{{\rm{p}}}}}}}({t}_{i+1})$ is the Contribution Ratio of Indoor Humidity at point $x$ and time ${t}_{i+1}$ (${{{{{{\rm{CRI}}}}}}}_{\left({{{{{\rm{H}}}}}}\right)}(x,{t}_{i+1})$). Then, the Rate of Humidity Contribution Change at point $x$ during the time $\triangle t={t}_{i+1}-{t}_{i}$ (${{{{{{\rm{CRI}}}}}}}_{\left({{{{{\rm{H}}}}}}\right)}^{{{{{{\rm{t}}}}}}}(x,{t}_{i},{t}_{i+1})$) can be obtained**. b** From Contribution Ratio of Indoor Climate (${{{{{{\rm{CRI}}}}}}}_{\left({{{{{\rm{C}}}}}}\right)}$) to Rate of Indoor Climate Contribution Change ${{{{{{\rm{CRI}}}}}}}_{({{{{{\rm{C}}}}}})}^{{{{{{\rm{t}}}}}}}$ with considering the dynamic variation per unit time. The temperature at a spatial point $x$ is $\theta (x)$ at $t=0$. After the heat source with heat flux ${\Theta }_{n}$ delivers the flow (air density ${\rho }_{{{{{{\rm{f}}}}}}}$, air flow rate ${V}_{{{{{{\rm{f}}}}}}}$) to the indoor environment, the temperature at point $x$ varies by $\delta \theta (x,{t}_{i})$ at $t={t}_{i}$ and $\delta \theta (x,{t}_{i+1})$ at $t={t}_{i+1}$ under actual situation, which varies by ${\theta }_{{{{{{\rm{p}}}}}}}({t}_{i})$ at $t={t}_{i}$ and ${\theta }_{{{{{{\rm{p}}}}}}}({t}_{i+1})$ at $t={t}_{i+1}$ under ideal situation (i.e. perfect mixing condition). The ratio of $\delta \theta (x,{t}_{i})$ to ${\theta }_{{{{{{\rm{p}}}}}}}({t}_{i})$ is the Contribution Ratio of Indoor Climate at point $x$ and time ${t}_{i}$ (${{{{{{\rm{CRI}}}}}}}_{\left({{{{{\rm{C}}}}}}\right)}(x,{t}_{i})$), and the ratio of $\delta \theta (x,{t}_{i+1})$ to ${\theta }_{{{{{{\rm{p}}}}}}}({t}_{i+1})$ is the Contribution Ratio of Indoor Climate at point $x$ and time ${t}_{i+1}$ (${{{{{{\rm{CRI}}}}}}}_{\left({{{{{\rm{C}}}}}}\right)}(x,{t}_{i+1})$). Then, the Rate of Indoor Climate Contribution Change at point $x$ during the time $\triangle t={t}_{i+1}-{t}_{i}$ (${{{{{{\rm{CRI}}}}}}}_{\left({{{{{\rm{C}}}}}}\right)}^{{{{{{\rm{t}}}}}}}(x,{t}_{i},{t}_{i+1})$) can be obtained.

${{{{{{\rm{CRI}}}}}}}_{\left({{{{{\rm{H}}}}}}\right)}$ refers to the ratio of the rise (or fall) in humidity at a point from an individual moisture source to the rise (or fall) in humidity under perfect mixing conditions for the same moisture source^30^ (Fig. 9a). It indicates the spatial distribution of humidity influenced by the moisture source. When the air humidity at the point is higher than the initial humidity, ${{{{{{\rm{CRI}}}}}}}_{\left({{{{{\rm{H}}}}}}\right)}$ is larger than 1.0 and vice versa.

6 $${{{{{{\rm{CRI}}}}}}}_{\left({{{{{\rm{H}}}}}}\right)}\left(x\right)=\frac{\delta X(x)}{{X}_{{{{{{\rm{p}}}}}}}}=\frac{\delta X(x)}{\frac{{q}_{n}}{{\rho }_{{{{{{\rm{f}}}}}}}\cdot {V}_{{{{{{\rm{f}}}}}}}}}$$

where $\delta X(x)$ is the rise (or fall) in humidity at a point $x$ due to the moisture source (g kg^−1^). ${X}_{{{{{{\rm{p}}}}}}}$ is the rise (or fall) in humidity under perfect mixing conditions due to the moisture source (g kg^−1^). Since perfect mixing conditions cannot be achieved due to experimental limitations, the difference between the average specific humidity at all measured points and the initial specific humidity at a point $x$ is taken to be ${X}_{{{{{{\rm{p}}}}}}}$ in this study. ${q}_{n}$ is the source intensity of the moisture source (g s^−1^).

Taking the time derivation of ${{{{{{\rm{CRI}}}}}}}_{\left({{{{{\rm{H}}}}}}\right)}$ (Fig. 9a), ${{{{{{\rm{CRI}}}}}}}_{({{{{{\rm{H}}}}}})}^{{{{{{\rm{t}}}}}}}$ can be obtained, which refers to the change rate of the moisture source contribution to the air humidity at point $x$ during the time $\triangle t={t}_{i+1}-{t}_{i}$. It reflects the dynamic characteristic of the humidity variation under the influence of the moisture source, in unit of s^−1^.

7 $${{{{{{\rm{CRI}}}}}}}_{({{{{{\rm{H}}}}}})}^{{{{{{\rm{t}}}}}}}\left(x,{t}_{i},{t}_{i+1}\right)=\mathop{{{{{{\rm{lim}}}}}}}\limits_{\triangle t\to 0}\frac{{{{{{{\rm{CRI}}}}}}}_{\left({{{{{\rm{H}}}}}}\right)}\left(x,{t}_{i+1}\right)-{{{{{{\rm{CRI}}}}}}}_{\left({{{{{\rm{H}}}}}}\right)}\left(x,{t}_{i}\right)}{{t}_{i+1}-{t}_{i}}$$

where ${{{{{{\rm{CRI}}}}}}}_{\left({{{{{\rm{H}}}}}}\right)}\left(x,{t}_{i}\right)$ is the contribution ratio of indoor humidity of the moisture source at point $x$, time ${t}_{i}$, and ${{{{{{\rm{CRI}}}}}}}_{\left({{{{{\rm{H}}}}}}\right)}\left(x,{t}_{i+1}\right)$ is that of the same point $x$ at time ${t}_{i+1}$.

### From ${{{{{{\bf{CRI}}}}}}}_{({{{{{\bf{C}}}}}})}$ to ${{{{{{\bf{CRI}}}}}}}_{({{{{{\bf{C}}}}}})}^{{{{{{\bf{t}}}}}}}$

${{{{{{\rm{C}}}}}}{{{{{\rm{RI}}}}}}}_{({{{{{\rm{C}}}}}})}$ refers to the ratio of temperature rise (or fall) at a point due to one individual heat source to the temperature rise (or fall) from the heat source uniformly distributed with the same amount of heat^28^ (Fig. 9b). It indicates the diffusion range of the heat generated by the heat source and its effect on the indoor temperature distribution.

8 $${{{{{{\rm{CRI}}}}}}}_{\left({{{{{\rm{C}}}}}}\right)}\left(x\right)=\frac{\delta \theta (x)}{{\theta }_{{{{{{\rm{p}}}}}}}}=\frac{\delta \theta (x)}{\frac{{\Theta }_{n}}{{c}_{{{{{{\rm{p}}}}}}}\cdot {\rho }_{{{{{{\rm{f}}}}}}}\cdot {V}_{{{{{{\rm{f}}}}}}}}}$$

where $\delta \theta (x)$ is the temperature rise (or fall) at a point $x$ due to the heat source (°C). ${\theta }_{{{{{{\rm{p}}}}}}}$ is the temperature rise (or fall) under perfect mixing conditions due to the heat source (°C). Due to the limitations of experimental conditions, the difference between the average air temperature of all measuring points and the initial temperature at point $x$ is taken as ${\theta }_{{{{{{\rm{p}}}}}}}$ in this study. ${\Theta }_{n}$ is the heat flux generated by the heat source (J s^−1^).

In Fig. 9b, similarly, ${{{{{{\rm{CRI}}}}}}}_{({{{{{\rm{C}}}}}})}^{{{{{{\rm{t}}}}}}}$ can be obtained by taking the time derivation of ${{{{{{\rm{C}}}}}}{{{{{\rm{RI}}}}}}}_{({{{{{\rm{C}}}}}})}$, which refers to the change rate of the heat source’s contribution to the air temperature at point $x$ from ${t}_{i}$ to ${t}_{i+1}$. It represents the variation of the temperature distribution per unit time under the influence of the heat source, in unit of s^−1^.

9 $${{{{{{\rm{CRI}}}}}}}_{({{{{{\rm{C}}}}}})}^{{{{{{\rm{t}}}}}}}\left(x,{t}_{i},{t}_{i+1}\right)=\mathop{{{{{{\rm{lim}}}}}}}\limits_{\triangle t\to 0}\frac{{{{{{{\rm{CRI}}}}}}}_{\left({{{{{\rm{C}}}}}}\right)}\left(x,{t}_{i+1}\right)-{{{{{{\rm{CRI}}}}}}}_{\left({{{{{\rm{C}}}}}}\right)}\left(x,{t}_{i}\right)}{{t}_{i+1}-{t}_{i}}$$

where ${{{{{{\rm{CRI}}}}}}}_{\left({{{{{\rm{C}}}}}}\right)}\left(x,{t}_{i}\right)$ is the contribution ratio of indoor climate of the heat source at point $x$, time ${t}_{i}$ and ${{{{{{\rm{CRI}}}}}}}_{\left({{{{{\rm{C}}}}}}\right)}\left(x,{t}_{i+1}\right)$ is that of the same point $x$ at time ${t}_{i+1}$.

## Data Availability

The data that support the findings of this study are available from the authors on reasonable request. Underlying data for the figures in the main manuscript is included as an excel file in source data.
